# Influence of psychostimulants and opioids on epigenetic modification of class III histone deacetylase (HDAC)-sirtuins in glial cells

**DOI:** 10.1038/s41598-021-00836-z

**Published:** 2021-10-29

**Authors:** Kalaiselvi Sivalingam, Mayur Doke, Mansoor A. Khan, Thangavel Samikkannu

**Affiliations:** Department of Pharmaceutical Sciences, Irma Lerma Rangel College of Pharmacy, Texas A&M University, 1010 W Avenue B, Kingsville, TX 78363 USA

**Keywords:** Neuroscience, Neurology

## Abstract

Substance abuse affects the central nervous system (CNS) and remains a global health problem. Psychostimulants, such as cocaine and methamphetamine (METH), and opioids affect neuronal function and lead to behavioral impairments via epigenetic modification. Epigenetic changes occur via classical pathways, especially the class III histone deacetylase (HDAC)-sirtuin (SIRT) family, that act as cellular sensors to regulate energy homeostasis and coordinate cellular responses to maintain genome integrity. However, SIRT family (1–7)-associated neurodegeneration has not been elucidated in the context of energy metabolism. The present study examined the effects of psychostimulants, such as cocaine and METH, and opioids, such as morphine, on SIRT family (1–7) [class I, II, III and IV] expression and cellular translocation-mediated dysfunction in astrocytes and microglial cells. The “nootropic” drug piracetam played a preventative role against psychostimulant- and opioid-induced SIRT (1–7) expression in astrocytes. These results indicate that cocaine, METH, and morphine affected deacetylation and cellular function, and these changes were prevented by piracetam in astrocytes.

## Introduction

Drug addiction is a major public health crisis worldwide, and the development of efficient medications for drug abuse treatment is a continuing major challenge. Substance use disorder (SUD) causes 11.8 million annual deaths, and approximately 9.2% of Americans used illicit drugs, especially cocaine, methamphetamine (METH) and morphine, within the past month^1,2^. These psychostimulants and opioids target several organs, including the central nervous system (CNS), and induce oxidative stress, reactive oxygen species (ROS) production, and metabolic dysfunction, including epigenetic modifications^3–5^. These effects ultimately lead to neuronal and behavioral impairments^6^.

Astrocytes and microglia are the predominant cell populations in the CNS and are influenced by psychostimulant-associated neuronal impairment^7,8^. Glial cells play an essential role in the CNS, including the supply of energy metabolites and neurotransmitters and protection from neuroinflammation^9^. Modulation of glial pathophysiological responses has beneficial effects on neurons injured by brain insults and neurodegenerative diseases, and glial cells are promising targets for neuroprotective drugs^10^. Cocaine, METH and morphine induced astrocyte activation and increased the expression level of the astrocyte marker glial fibrillary acidic protein (GFAP) in vitro and in the rodent brain^11–13^. Drugs of abuse activate microglia, which further affects cellular function and influence neuronal disorders^14^.

Cocaine, METH and morphine ultimately impact several cellular functions, including energy metabolism and epigenetic modifications in the CNS^5,15^. Epigenetic silencing and inappropriate recruitment initiate and aggravate many different diseases. Previous studies documented that epigenetic modifications and recruitment in the molecular processes led to addiction to psychostimulants and opioids^16^. Cocaine, METH and morphine also impact epigenetic changes in DNMTs, HDACs, HAD/HAT protein and miRNA expression, which lead to neuronal functions^17–19^.

The current study primarily focused on the impact of psychostimulants and opioids on histone deacetylases. Sirtuins (SIRTs 1–7) [classes I, II, III and IV] are nicotinamide adenine dinucleotide (NAD^+^)-dependent class III HDACs. NAD^+^ is a central metabolic cofactor in eukaryotic cells that plays a critical role in regulating cellular metabolism and energy homeostasis of the cell. SIRTs are divided into four different classes (I-IV). Class I SIRTs-1, 2 and 3 have strong deacetylase activity in the presence of NAD^+^, and class II SIRT-4 has ADP-ribosyltransferase activity. Class III SIRT-5 has NAD-dependent demalonylase, desuccinylase and deacetylase activity. Class IV SIRTs-6 and 7 are substrate-specific deacetylase enzymes, and SIRT-6 also exhibits ADP-ribosyl transferase activity^20^. The different classes of SIRTs are localized in various subcellular regions to regulate a wide variety of cellular functions.

The dysregulation/overexpression of mammalian SIRT activity disturbs various biological processes, including cellular metabolism and the deacetylation of histone and nonhistone proteins^19^. Sirtuins have wide-ranging effects on metabolic homeostasis, primarily via their role as master regulators of mitochondrial integrity. Cocaine and morphine alter SIRT-1 expression^21^. A recent report provides significant support for the importance of SIRTs in many biological processes, and there is broad interest in developing SIRT-regulating drugs^22^.

Piracetam (2-oxo-1-pyrrolidine acetamide) is a cyclic derivative of the neurotransmitter γ-aminobutyric acid (GABA), and it is commonly called a “nootropic” drug (i.e., a drug that modulates cognitive function without causing sedation or stimulation^23^), which helps treat cognitive impairment in aging and dementia patients^24^. Piracetam reduced neuronal loss and increased hippocampal synapses in alcohol-treated rats and acted as an anticonvulsant drug to improve neural plasticity^25^.

Piracetam exerted a protective effect against lipopolysaccharide (LPS)-induced neuroinflammation^26^ and improved neuritogenesis in the human neuronal cell line SH-SY5Y^27^. However, some adverse effects of piracetam were reported^28^. Piracetam is a clinically known drug, but its protective effects against psychostimulant-induced neuronal dysfunction were not examined. Well-recognized nootropic drugs are used for the treatment of various psychiatric disorders^29^. Neuroprotective agents and analogs with psychostimulant drug effects may reduce neuronal toxicity and prevent or protective neuronal functions^30–32^. Our recent studies revealed that piracetam prevented mitochondrial and nuclear epigenomic integrity and cocaine-induced hypomethylation in astrocytes^33^. The present study investigated the effects of cocaine-, METH- and morphine-mediated induction of SIRT (1–7) expression in astrocytes and microglia and analyzed the preventive effects of piracetam on psychostimulant- and opioid-induced SIRT expression in astrocytes.

## Results

### Differential expression of SIRTs in astrocytes and microglia exposed to cocaine, METH and morphine

**Figure 1:**
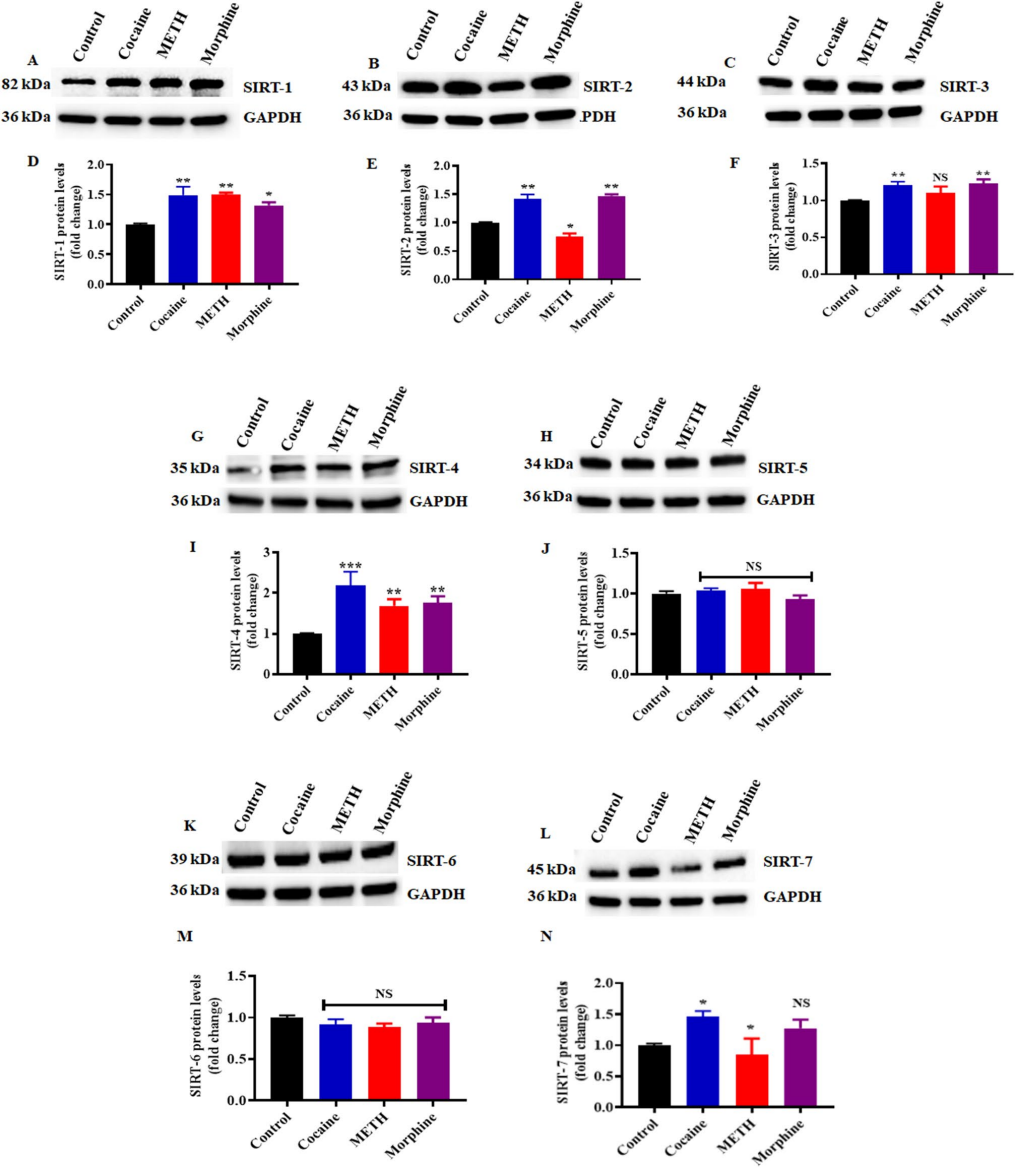
Effects of psychostimulants and opioids on SIRTs (1–7) in human primary astrocytes. Cells were exposed to cocaine (1 µM), METH (10 µM) and morphine (5 µM) for 24 h. The protein expression levels of different classes of SIRTs (1–7) in astrocytes were determined using Western blotting analysis with GAPDH as a loading control. Western blot showing (**A**) SIRT-1, (**B**) SIRT-2, (**C**) SIRT-3, (**G**) SIRT-4, (**H**) SIRT-5, (**K**) SIRT-6 and (**L**) SIRT-7. Densitometric analyses (**D**–**F**,**I**,**J**,**M**,**N**) represent the protein levels (fold-change from control). The data are expressed as the means ± SD of three independent experiments. ***P < 0.001, **P < 0.01, *P < 0.05, NS—Nonsignificant. Treatments groups were compared to Control. The supplementary information includes all full-length blots.

The present study determined whether the psychostimulant drugs cocaine and METH and the opioid morphine induced similar or distinct mechanisms of SIRT (1–7) expression in astrocytes and microglia. To examine the effects of cocaine, METH and morphine on SIRT expression (1–7), cells were exposed to cocaine (1 µM), METH (10 µM), and morphine (5 µM) for 24 h. The exposure of astrocytes to psychostimulants and opioids significantly increased SIRT-1, SIRT-2, SIRT-3, and SIRT-4 (Fig. 1A–G,I), and METH exposure significantly decreased the expression of SIRT-2 (Fig. 1B,E). There was no significant increase in SIRT-3 expression (Fig. 1C,F). Cocaine, METH and morphine did not cause any significant changes in SIRT-5 (Fig. 1H,J) or SIRT-6 expression (Fig. 1K,M) compared to the control. Cocaine increased the expression of SIRT-7, while METH decreased the expression of SIRT-7.Gowever, morphine induced no changes (Fig. 1L,N).

**Figure 2:**
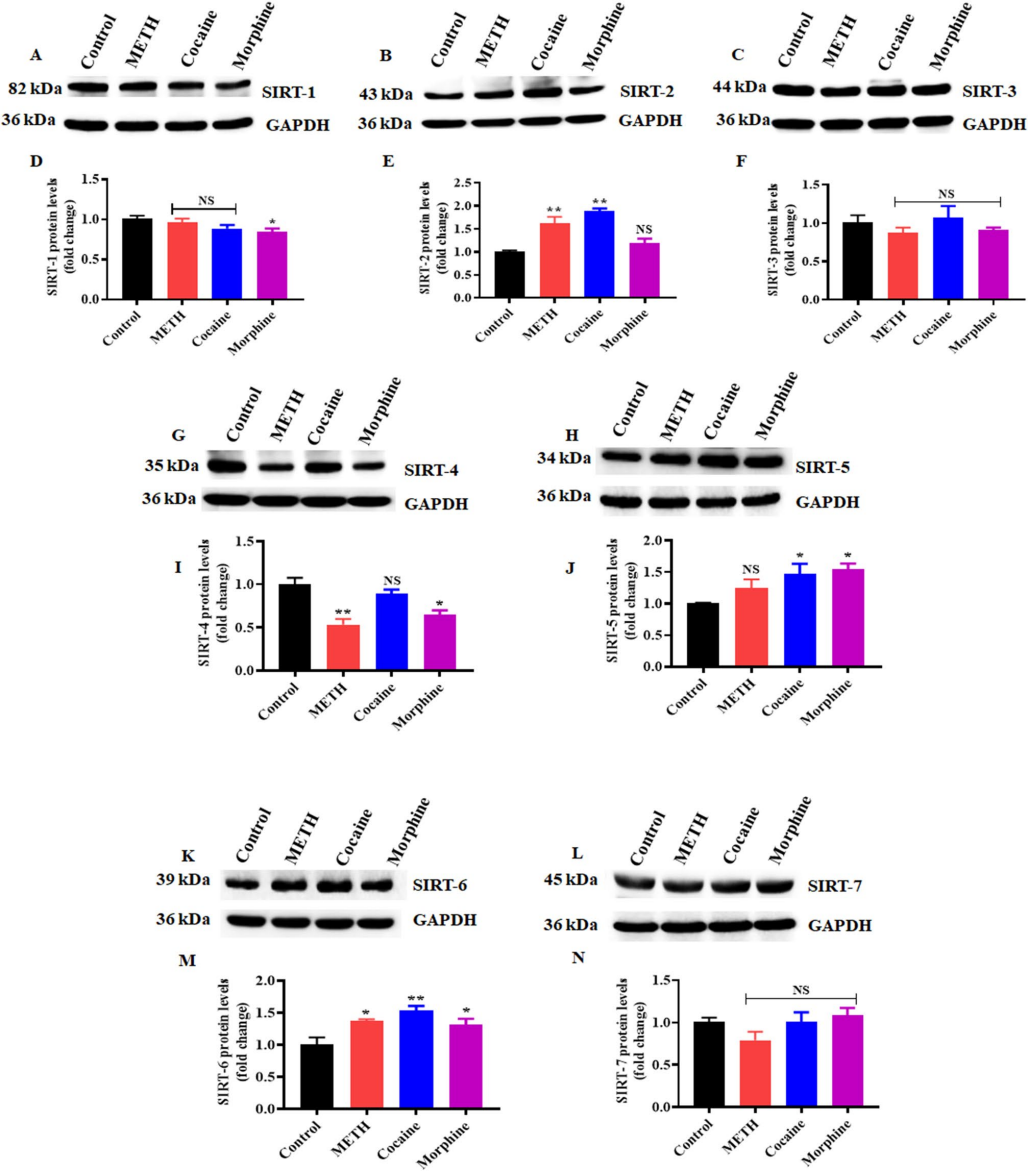
Effects of psychostimulants and opioids on SIRTs (1–7) in human primary microglia. Cells were exposed to METH (5 µM), cocaine (1 µM), and morphine (1 µM) for 24 h. The protein expression levels of different classes of SIRTs (1–7) in the microglia were determined using Western blotting analysis with GAPDH as a loading control. Western blot showing (**A**) SIRT-1, (**B**) SIRT- 2, (**C**) SIRT-3, (**G**) SIRT-4, (**H**) SIRT-5, (**K**) SIRT-6 and (**L**) SIRT-7. Densitometric analyses (**D**–**F**,**I**,**J**,**M**,**N**) represent the protein levels (fold-change from control). The data are expressed as the means ± SD of three independent experiments. ***P < 0.001, **P < 0.01, *P < 0.05, NS—Nonsignificant. Treatments groups were compared to Control. The supplementary information includes all full-length blots.

We examined the impact of cocaine, METH and morphine-induced differential expression of SIRTs in human brain immune cell microglia. Cells were exposed to METH (5 µM), cocaine (1 µM), and morphine (1 µM) for 24 h. Exposure of microglia to these drugs significantly increased SIRT-2, SIRT-5, and SIRT-6 expression (Fig. 2B,E,H,J,K,M) and downregulated SIRT-4 expression (Fig. 2G,I). However, there were no significant changes in SIRT-1, SIRT-3, or SIRT-7 (Fig. 2A,C,D,F,L,N) compared with the control, but morphine significantly reduced the level of SIRT-1 expression (Fig. 2A,D) when compared with control. We found that exposure of microglia to these drugs caused differential SIRT expression compared to astrocytes. The activation of SIRT expression in each cell depends on cellular functions and the stress response^34^.

### The psychostimulants cocaine and METH and the opioid morphine affect SIRT gene expression in astrocytes, and piracetam plays a protective role

**Figure 3:**
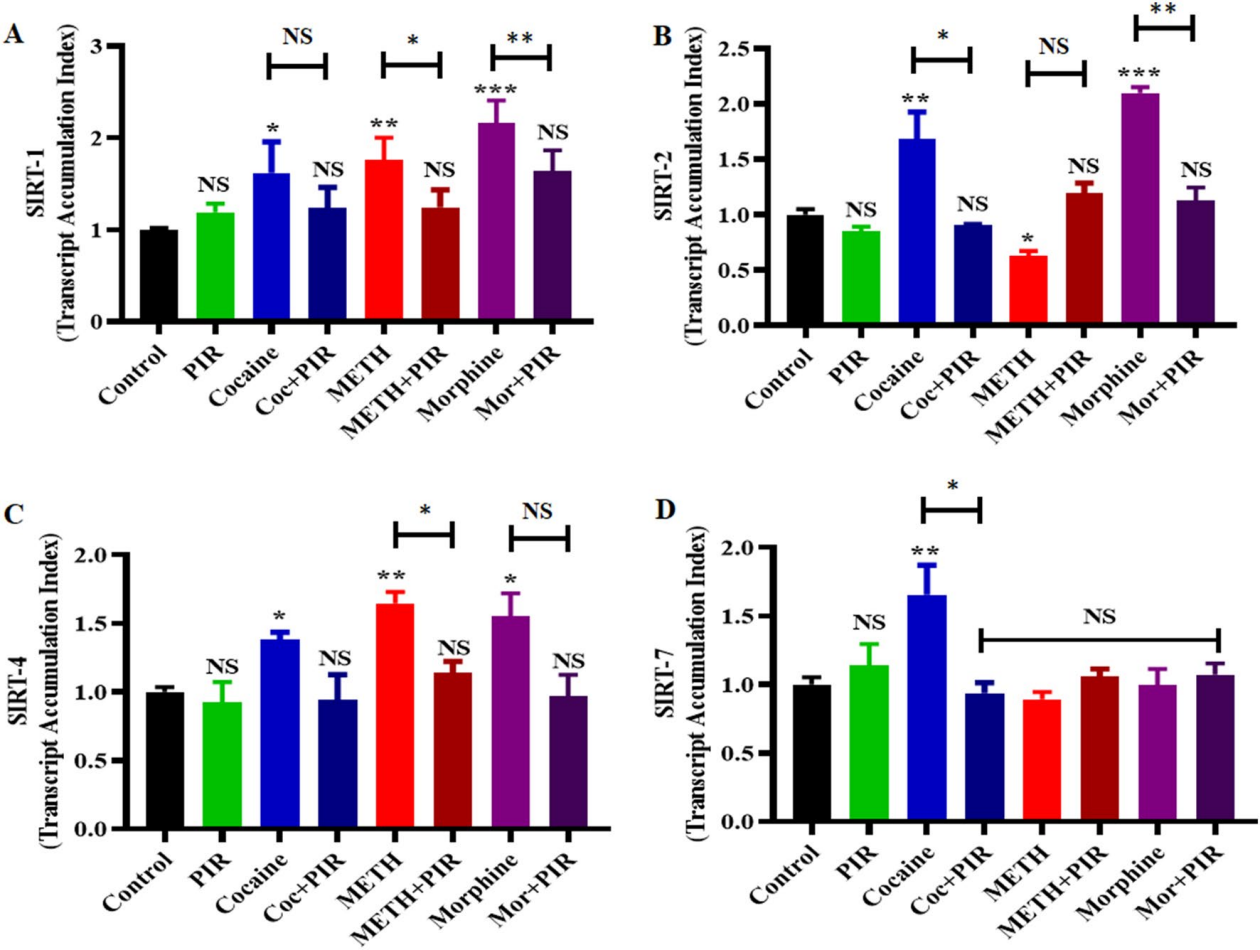
Piracetam reversed the effects of psychostimulants and opioids on the differential gene expression of SIRTs. Human primary astrocytes were exposed to cocaine (1 µM), METH (10 µM) or morphine (5 µM) alone or in combination with piracetam (10 µM) for 24 h. Controls were maintained in drug-free medium (without drug exposure). (**A**) SIRT-1, (**B**) SIRT-2, (**C**) SIRT-4, and (**D**) SIRT-7 mRNA expression levels in astrocytes determined using qRT-PCR analysis with the housekeeping gene β-actin as a loading control. The data are expressed as the means ± SD of the transcript accumulation index (TAI) (compared to control) of three independent experiments. ***P < 0.001, **P < 0.01, *P < 0.05, NS—Nonsignificant. Coexposure of psychostimulant or opioid with piracetam compared to psychostimulant or opioid alone.

To determine the protective effects of piracetam on the deacetylating SIRT enzymes, astrocytes were exposed to cocaine (1 µM), METH (10 µM) and morphine (5 µM) alone and in combination with piracetam (10 µM) for 24 h. Figure 3 shows that exposure to cocaine and morphine upregulated the mRNA expression of SIRT-1, SIRT-2, and SIRT-4 (Fig. 3A–C), and METH exposure upregulated only SIRT-1 and SIRT- 4 (Fig. 3A,C) and downregulated the expression of SIRT-2 (Fig. 3B). Cocaine increased SIRT-7 mRNA expression, and METH and morphine exposure did not induce any changes (Fig. 3D) when compared with control. Notably, coexposure of the drugs with piracetam prevented these mRNA expression levels in a drug-dependent manner (Fig. 3A–D). These results suggest that the protective effects of piracetam help counteract the impact of cocaine, METH and morphine on deacetylation activity in primary astrocytes.

### The psychostimulants cocaine and METH and the opioid morphine affect class I SIRT protein modification, and piracetam plays a protective role

**Figure 4:**
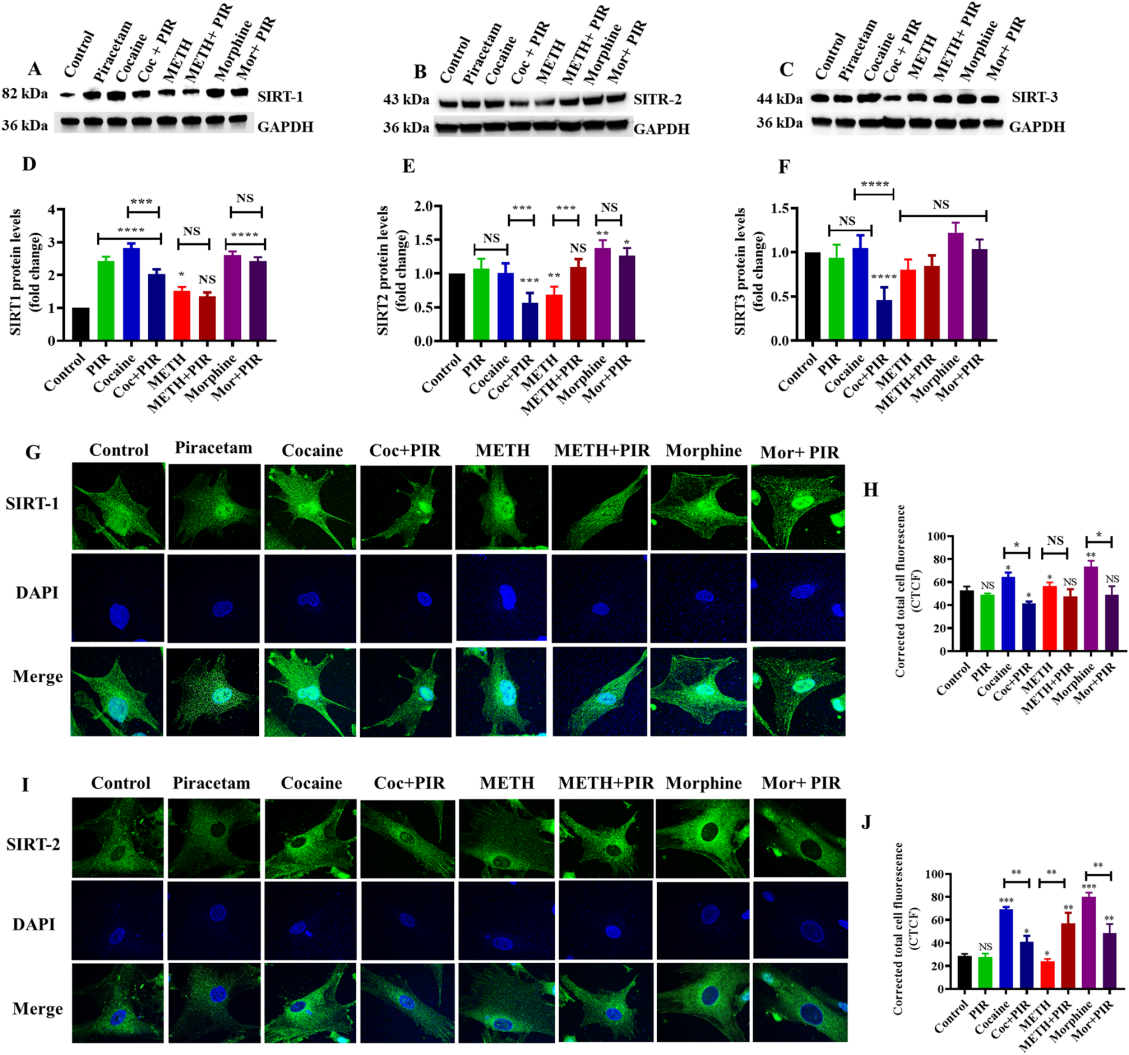
The protective effect of piracetam against the effects of psychostimulants and opioids on class I SIRT proteins. Human primary astrocytes were exposed to cocaine (1 µM), METH (10 µM) and morphine (5 µM) alone or in combination with piracetam (10 µM) for 24 h. Controls were maintained in drug-free medium (without drug exposure). Representative blots showing the expression of class I sirtuins (**A**) SIRT-1, (**B**) SIRT-2, and (**C**) SIRT-3. (**D**–**F**) Densitometric analyses of each protein level to its corresponding GAPDH loading control (fold-change from control). (**G**) Effects of psychostimulants and opioids on the expression and translocation of SIRT-1 in the cytoplasm and nucleus. SIRT-1 expression (green) and nuclear staining with DAPI (blue) were observed using confocal microscopy (magnification 100$\times$, scale bar 100 μm). (**H**) Quantification of SIRT-1 fluorescence intensity (CTCF). (**I**) Effect of psychostimulants and opioids on the expression pattern of SIRT-2 in the cytoplasm. SIRT-2 expression (green) and nuclear staining with DAPI (blue) were observed using confocal microscopy (magnification 100x, scale bar 100 μm). (**J**) Quantification of SIRT-2 fluorescence intensity (CTCF). Data are expressed as the means ± SD of three independent experiments. ***P < 0.001, **P < 0.01, *P < 0.05, NS—Nonsignificant. Coexposure of psychostimulant or opioid with piracetam compared to psychostimulant or opioid alone. The supplementary information includes all full-length blots.

We also examined the effects of cocaine, METH and morphine on SIRT-1, SIRT-2 and SIRT-3 protein expression and the protective effects of piracetam in astrocytes. We found that cocaine and morphine exposure upregulated SIRT-1 (F(7,14) = 58.94, P < 0.0001) and SIRT-2 (F(7,14) = 31.55, P = 0.0013) (Fig. 4A–F) protein expression. METH exposure upregulated SIRT-1 (F(7,14) = 58.94, P = 0.0126) (Fig. 4A,D) and downregulated SIRT-2 (F(7,14) = 31.55, P = 0.0076) (B and E) but caused no significant changes in SIRT-3 (Fig. 4C,F) when compared with control. These results confirmed similar patterns to the mRNA expression. Notably, coexposure with piracetam prevented the effects of cocaine, METH and morphine on SIRT-1, SIRT-2 and SIRT-3 protein expression in astrocytes (Fig. 4A–F).

### The psychostimulants cocaine and METH and the opioid morphine affect the subcellular localization of SIRT-1 and SIRT-2 in astrocytes

SIRT-1 and SIRT-2 are localized in the cytoplasm and nucleus, respectively. Psychostimulants and opioid exposure may impact these proteins via crosstalk or translocation and lead to cellular dysfunction. The present study performed immunostaining analysis to confirm the cytoplasmic and nuclear localization of SIRT-1 and SIRT-2 in astrocytes. The results showed that SIRT-1 primarily existed in the nucleus or cytoplasm in cocaine-, METH- and morphine-exposed cells. However, cocaine, METH and morphine treatment with piracetam markedly altered SIRT-1 nuclear localization and total expression in astrocytes (Fig. 4G,H). The nuclear translocation of the metabolic sensor SIRT-1 may help regulate energy homeostasis in response to transcription factors. However, SIRT-1 deacetylase activity enhances NAD^+^ concentration during energy reduction, which plays a critical role in glycolysis and cellular respiration for ATP production.

Mammalian SIRT-2 is predominantly present in the cytoplasm, but it shuttles between the cytoplasm and nucleus during G2/M transition and deacetylates histone H4 at lysine 16 to modulate chromatin condensation during metaphase. Figure 4 shows that cocaine and morphine upregulated the expression of SIRT-2, and METH decreased its expression in the cytoplasm. Piracetam prevented these effects in drug-treated cells (Fig. 4I,J). SIRT-2 is associated with lysine acetylation/deacetylation in the regulation of energy homeostasis. However, the neuroprotective drug piracetam prevented SIRT-2 overexpression in the cytoplasm in astrocytes.

### The psychostimulants cocaine and METH and the opioid morphine affect mitochondrial proteins, and piracetam protected SIRT-3 colocalization

**Figure 5:**
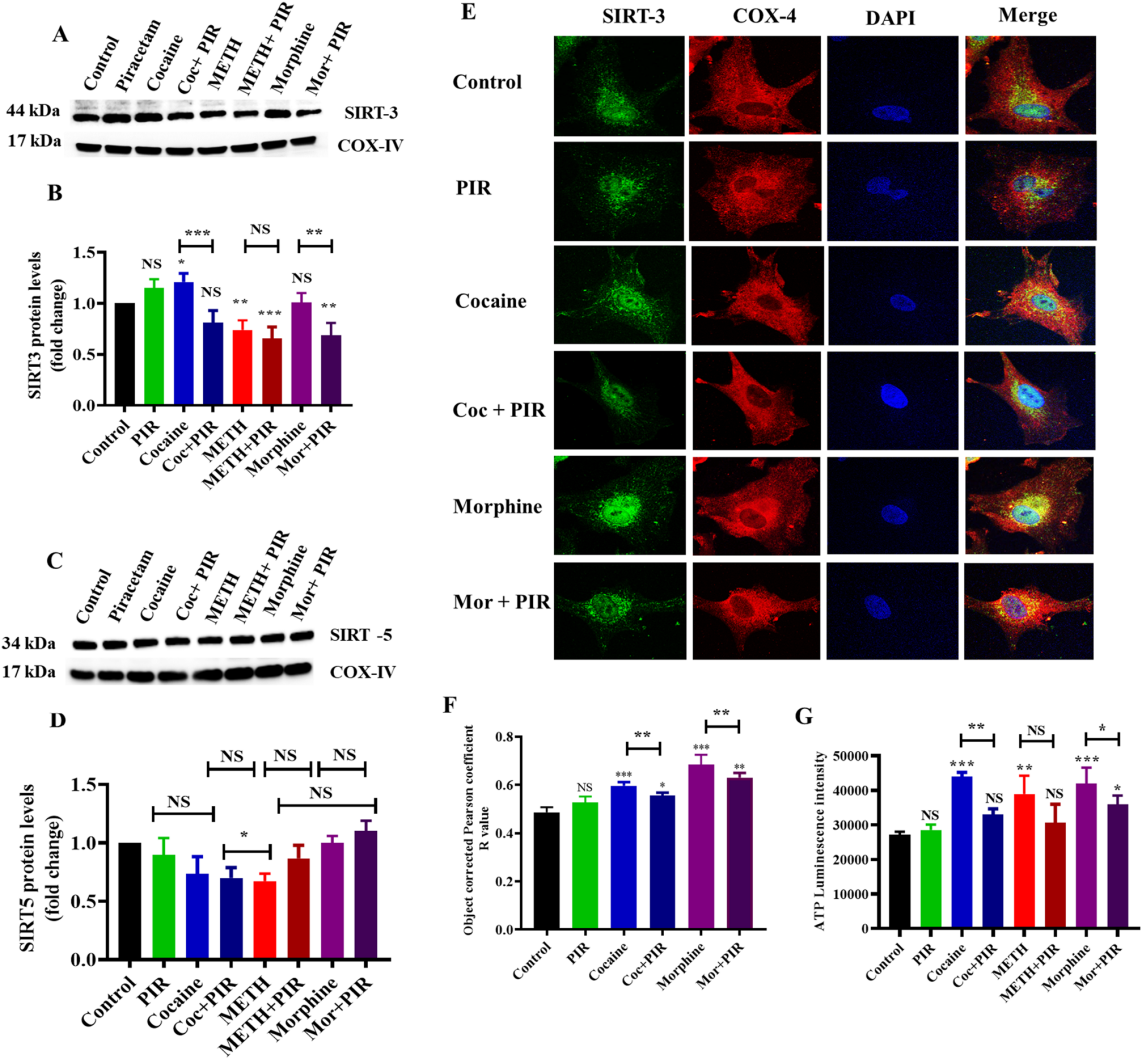
The protective effects of piracetam against the effects of psychostimulants and opioids on the mitochondrial protein expression of SIRT-3 and SIRT-5. Human primary astrocytes were exposed to cocaine (1 µM), METH (10 µM) and morphine (5 µM) alone or in combination with piracetam (10 µM) for 24 h to isolate the mitochondrial fraction. Controls were maintained in drug-free medium (without drug exposure). Representative blots showing the expression of mitochondrial proteins (**A**) SIRT-3 and (**C**) SIRT-5. (**B**) and (**D**) Densitometric analyses of each protein level to its corresponding COX-IV loading control (fold-change from control). Data are expressed as the means ± SD of three independent experiments. ***P < 0.001, **P < 0.01, *P < 0.05, NS—Nonsignificant. (**E**) Effects of psychostimulants and opioids on SIRT-3 expression and colocalization. SIRT-3 expression (green), mitochondrial membrane protein COX-IV (red) and nuclear stain DAPI (blue) were observed using confocal microscopy (magnification 100x, scale bar 100 μm). (**F**) Quantification of SIRT-3 colocalization with the mitochondrial membrane protein COX-IV was analyzed using Pearson’s coefficient r value in three different regions of interest (ROIs) for the same cell. (**G**) Effects of psychostimulants and opioids on intracellular ATP content. Data are expressed as the means ± SD of luminescent intensity in triplicate. ***P < 0.001, **P < 0.01, *P < 0.05, NS—Nonsignificant. Coexposure of psychostimulant or opioid with piracetam compared to psychostimulant or opioid alone. The supplementary information includes all full-length blots.

Acetylation/deacetylation is a key regulator of mitochondrial metabolism and cellular function^35^. Mitochondrial proteins are deacetylated by NAD^+^-dependent SIRT deacetylases, which are key regulators of mitochondrial integrity via the maintenance of the metabolic and redox balance in the mitochondria under physiological and pathological conditions^36^. Mitochondrial SIRT-3, -4 and -5 participate in the regulation of ATP production, metabolism and cell signaling. SIRT-3 deacetylates and activates mitochondrial enzymes involved in fatty acid β-oxidation, amino acid metabolism and the electron transport chain (ETC), which lead to increased mitochondrial energy metabolism and ATP production^34,37^. Drug abuse has devastating effects and leads to neuroadaptations that require more energy for the reward system. To assess the possible protective effect of piracetam on specific subcellular localization, we analyzed the expression of SIRT-3 and SIRT-5 in mitochondria. We found that cocaine (F(7,14) = 28.34, P = 0.0363) exposure upregulated mitochondrial SIRT-3 protein expression, but there were no significant changes in SIRT-5 expression (Fig. 5A–D). METH-exposed cells showed no significant changes in SIRT-3, but SIRT-5 (F(7,14) = 8.285, P = 0.0363) expression was downregulated (Fig. 5A–D) compared to the control. Coexposure to these drugs and piracetam prevented the effects on mitochondrial SIRT-3 and SIRT-5 protein expression in astrocytes (Fig. 5A–D).

Immunostaining was performed to validate our protein expression analysis of SIRT-3. Notably, SIRT-3 was localized exclusively in the mitochondria and nucleus. However, the histone deacetylase activity of SIRT-3 in the nucleus was not elucidated. The results of the present study confirmed that exposure to cocaine and morphine increased the expression and mitochondrial colocalization of SIRT-3 in astrocytes (Fig. 5E,F). However, piracetam prevented the cocaine- and morphine-mediated overexpression of SIRT-3, which may protect mitochondrial biogenesis and regulate energy production (Fig. 5E,F). We also analyzed the total intracellular ATP content in cells treated with cocaine, METH and morphine, which significantly increased ATP levels in astrocytes. Piracetam prevented the impact of drugs of abuse on ATP levels in astrocytes (Fig. 5G).

### The psychostimulants cocaine and METH and the opioid morphine affect class II, III and IV SIRT protein modifications, and piracetam plays a protective role

**Figure 6:**
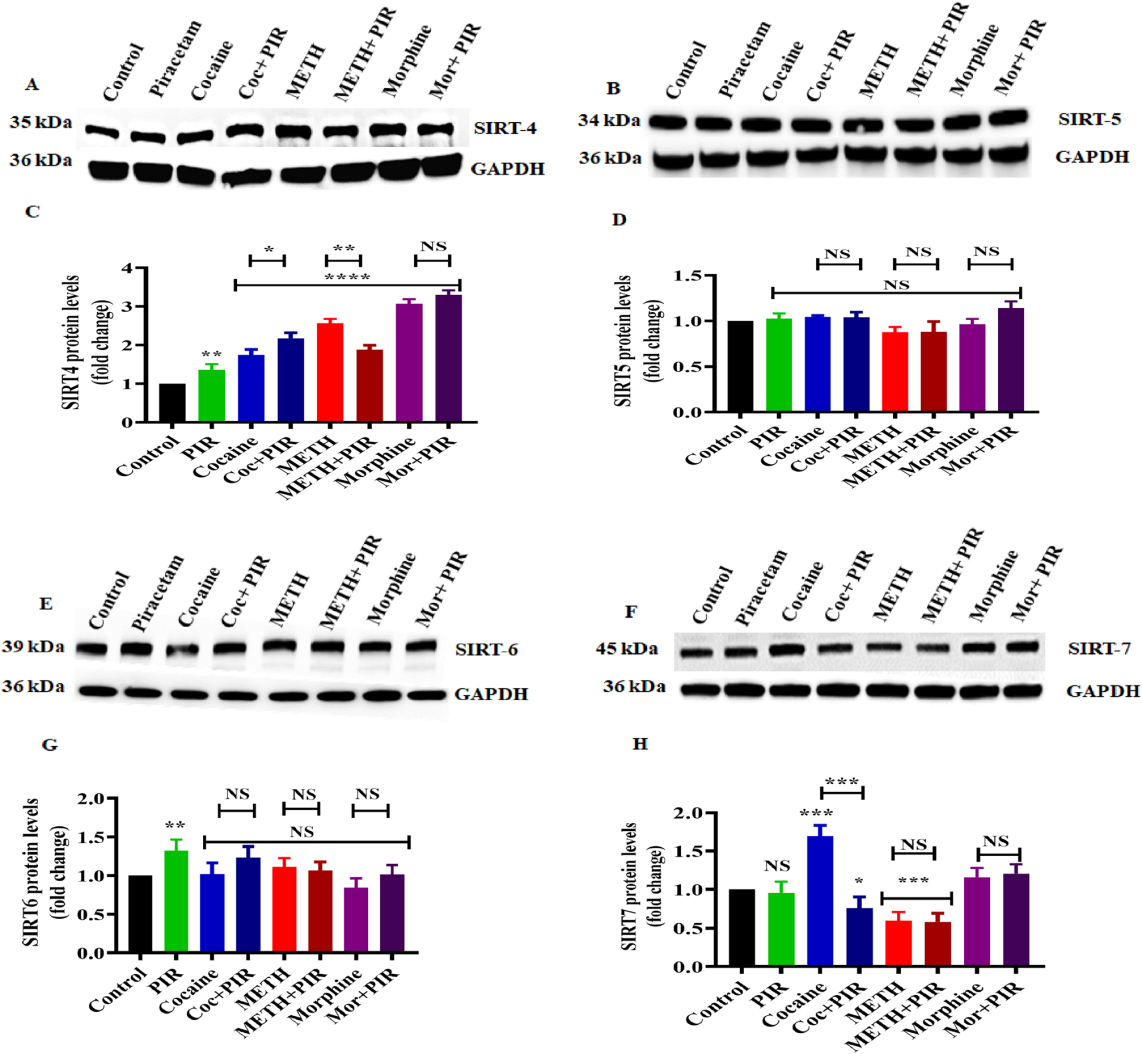
The protective effects of piracetam against psychostimulants and opioids on class II-IV SIRT protein expression in human astrocytes. Cells were exposed to cocaine (1 µM), METH (10 µM) and morphine (5 µM) alone or in combination with piracetam (10 µM) for 24 h. Controls were maintained in drug-free medium (without drug exposure). Representative blots showing the expression of (**A**) SIRT-4, (**B**) SIRT-5, (**E**) SIRT-6, and (**F**) SIRT-7. (**C**), (**D**), (**G**) and (**H**) Densitometric analyses of each protein level to its corresponding GAPDH loading control (fold-change from control). Data are expressed as the means ± SD of three independent experiments. ***P < 0.001, **P < 0.01, *P < 0.05, NS—Nonsignificant. Coexposure of psychostimulant or opioid with piracetam compared to psychostimulant or opioid alone. The supplementary information includes all full-length blots.

Figure 6 shows that exposure to cocaine, METH, and morphine increased SIRT-4 (F(7,14) = 275.6, P < 0.0001) protein expression (Fig. 6A,C). Notably, no significant changes were observed in SIRT-5 and SIRT-6 levels (Fig. 6B,D,E,G). Cocaine significantly increased SIRT-7 (F(7,14) = 57.6, P < 0.0001) modification, but no changes occurred in response to METH or morphine exposure (Fig. 6F,H) when compared with control. Coexposure of cocaine, METH and morphine with piracetam markedly prevented the protein modifications of SIRT-4 and SIRT-7 levels in a drug-dependent manner (Fig. 6A–H).

### The psychostimulants cocaine and METH and the opioid morphine affect the nuclear localization of SIRT-1 and SIRT-7, and piracetam plays a protective role

**Figure 7:**
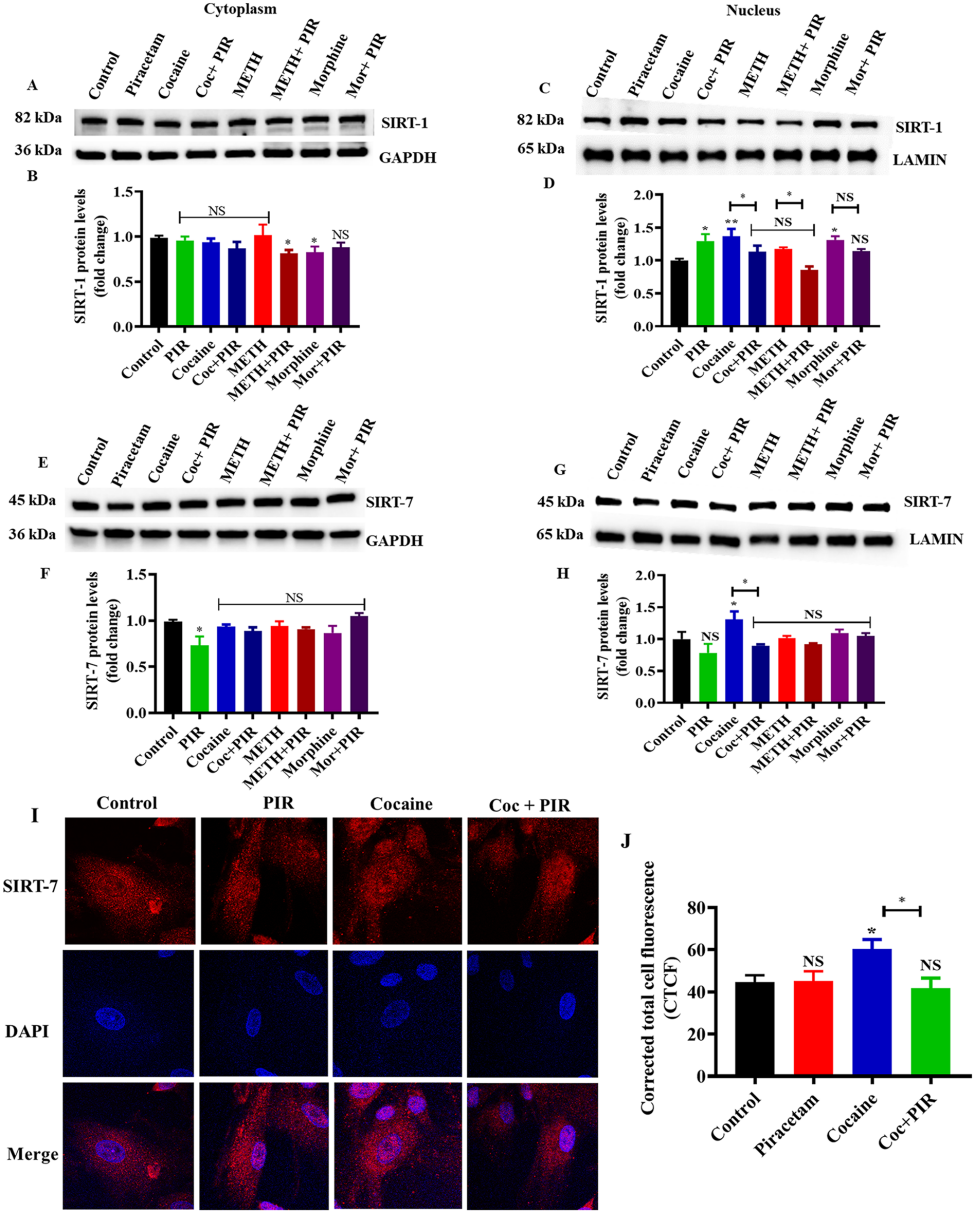
The protective effects of piracetam against the impact of psychostimulants and opioids on the cytoplasm and nuclear SIRT-1 and SIRT-7 protein expression. Human primary astrocytes were exposed to cocaine (1 µM), METH (10 µM) and morphine (5 µM) alone or in combination with piracetam (10 µM) for 24 h to isolate the nuclear fraction. Controls were maintained in drug-free medium (without drug exposure). Representative blots showing the expression of cytofractions (**A**) SIRT-1 and (**E**) SIRT-7 and nuclear fractions (**C**) SIRT-1 and (**G**) SIRT-7. (**B**,**D**,**F**,**H**) Densitometric analyses of each protein level to its corresponding loading control GAPDH and LAMIN (fold-change from control). (**I**) Effects of the psychostimulant cocaine on the expression and translocation of SIRT-7. SIRT-7 expression (red) and nuclear staining with DAPI (blue) were observed using confocal microscopy (magnification 100x, scale bar 100 μm). (**J**) Quantification of SIRT-7 fluorescence intensity (CTCF). Data are expressed as the means ± SD of three independent experiments. **P < 0.01, *P < 0.05, NS—Nonsignificant. Coexposure of psychostimulant or opioid with piracetam compared to psychostimulant or opioid alone. The supplementary information includes all full-length blots.

SIRT-1 and SIRT-7 are localized in the cytoplasm and nucleus. The SIRT-7 mechanism in the CNS is not well established, but it likely has major functions in neuronal pathways and diseases. We found that SIRT-1 (Fig. 7C,D) and SIRT-7 (Fig. 7G,H) were overexpressed in the cocaine-exposed nuclear fraction, but morphine only upregulated SIRT-1 (Fig. 7C,D). There were no significant changes in SIRT-1 or SIRT-7 in the METH-exposed nuclear fraction. Notably, no changes occurred in the expression levels of SIRT-1 (Fig. 7A,B) or SIRT-7 (Fig. 7E,F) in the cytoplasm, which may act as a transcription regulator in the nucleus. Combined exposure to piracetam prevented cocaine-induced SIRT-1 and SIRT-7 and morphine-induced SIRT-1 overexpression in the nuclei of astrocytes (Fig. 7C,D,G,H).

Immunostaining analysis was performed to confirm the cocaine-mediated overexpression and cellular localization of SIRT-7 in astrocytes. The results suggested the presence ofSIRT-7 in the cytoplasm and nucleus. Nuclear localization was substantially higher in cocaine-exposed cells than control cells. Piracetam regulated SIRT-7 expression and nuclear localization (Fig. 7I,J).

## Discussion

The psychostimulants cocaine and METH and the opioid morphine are associated with several pathological dysfunctions in the CNS and behavioral and neurocognitive impairments^38,39^. These drugs dysregulate cellular functions and induce oxidative stress, ROS production-induced alterations in pro-inflammation, mitochondrial biogenesis and epigenetic changes^39^. SIRT inhibition and expression were demonstrated in the brain. However, psychostimulants, such as cocaine and METH, and opioids, such as morphine, induced epigenetic modifications of class III HDAC-SIRTs (1–7) in the brain, especially in astrocytes and microglia, but the roles of these enzymes are not clear. The SIRT (1–7) signaling cascade plays a vital role in the neurodegeneration and/or neuroprotective pathways, which are cell- and drug-dependent^40^. Activation or destruction of SIRT deacetylase activity has profound biological consequences on mitochondrial biogenesis and oxidative energy metabolism in the CNS^41^. Growing evidence suggests that specific SIRTs regulate different neurodegenerative pathways, such as the metabolic switch of energy transfer, and mitochondrial dysregulation was linked to a number of neurodegenerative diseases, including Alzheimer’s and Parkinson’s diseases, and leads to psychiatric disorders, such as depression, addiction and mood disorders^42–45^. SIRT-1 positively regulates neuroprotection, and SIRT-3 is associated with traumatic brain injury (TBI). SIRT-3 is also involved in the neuroprotective effects of viniferin^20,46^.

The disruption of SIRT deacetylase activity in glial cells likely affects gene and protein expression to ultimately affect CNS energy homeostasis. Few studies reported the distribution and functional role of SIRTs (1–7) in the CNS^47^. Glial cells play a significant role in attenuating inflammation-mediated signals under physiological and pathological conditions. Subsequent reports revealed that SIRT expression was induced in various biological pathways related to oxidative stress, mitochondrial dysfunction, and inflammatory processes leading to neurodegenerative diseases^48^. Decreased levels of SIRT-1 in microglia contribute to cognitive decline and neuroinflammation^49^. Knockdown of SIRT2 in vivo and in vitro enhanced microglial activation and its associated proinflammation. The restoration of SIRT-3 expression in microglia reduced the apoptotic rate via promotion of mitochondrial functions and suppression of mitochondrial apoptosis^50^. However, the impact of drug abuse on the expression of SIRTs (1–7) in microglia was not elucidated. The results of the present study confirm that the differential expression of SIRTs (1–7) in astrocytes and microglia is cell-dependent based on their signaling pathways (Figs. 1 and 2).

Previous in vivo studies demonstrated that the repeated use of a psychostimulant drug, such as cocaine, induced overexpression of SIRT-1 and SIRT-2, and opioids, such as morphine, caused an overexpression of SIRT-1 in the nucleus accumbens (NAc), which is associated with excitability and long-lasting changes in the brain’s reward circuitry^42,51^. Acute exposure to cocaine does not alter SIRT-1 expression, which suggests that the upregulation of sirtuins in the NAc may contribute to the chronic neuroadaptations involved in drug addiction^52^. Exposure to psychostimulants and opioids for 12 h did not cause significant changes in SIRT expression in astrocytes in the present study (data not shown). The exposure of rats to foot shock and the self-administration of METH decreased the mRNA expression of SIRTs-2, 3, 4 and 5 in the dorsal striatum^53^. SIRT-2 is most abundant type in all brain regions, and particularly high levels are observed in myelin-producing oligodendrocytes (OLs)^54^. Chronic administration of METH increases striatal SIRT-1 and SIRT-2 expression^55^. Cocaine and morphine increased SIRT-1 and SIRT-2, and METH upregulated SIRT-1 and downregulated SIRT-2 in astrocytes (Fig. 1). The findings of the current study confirmed that cocaine, METH and morphine exposure increased SIRT-1 and SIRT-2 mRNA expression (Fig. 3) and protein modifications in the total and nuclear fractions compared to the control, but METH decreased SIRT-2 expression (Figs. 4 and 7). The neuroprotective drug piracetam prevented these notable changes in astrocytes.

NAD^+^-dependent SIRT deacetylase activity increases in cells exposed to cellular stress and calorie restriction, such as glucose, amino acid and nutrient depletion conditions, which increase NAD^+^ levels and produce more ATP via oxidative phosphorylation^56–58^. Mitochondrial sirtuins (mtSIRTs) are critical for the maintenance of cellular energy status via regulation of mitochondrial oxidative metabolism and protection of cells against the oxidative stress responses in the CNS^59^. Mitochondrial SIRT-3 deacetylates and activates acetyl-CoA synthetase 2 (ACS2), which helps convert free acetate to acetyl-CoA in the citric acid cycle to produce energy^60^. Mitochondrial SIRT-3 and SIRT-4 regulate ATP homeostasis via two interconnected mechanisms. SIRT-3 increases ATP production by deacetylating ETC enzymes, which increases respiration^34,37^, and SIRT-4 initiates a forward signaling response from mitochondria to the nucleus to regulate ATP levels^61^. Cocaine and morphine significantly upregulated SIRT-3 and SIRT-4 in the total and mitochondrial fractions in astrocytes compared to the control, and piracetam prevented these effects (Figs. 4, 5 and 6). Exposure to psychostimulants and opioids increases total ATP in astrocytes. Because psychostimulant drugs require substantial amounts of energy for the brain reward system, a shortage of metabolic supply develops, and increased SIRT activity helps satisfy the energy demand in the brain. Previous studies showed that METH exposure significantly affected mitochondrial proteins and enzymatic activities in animal models^62,63^. Addition METH also caused significant decreases in SIRT-5 mRNA levels, but there were no significant changes in SIRT-3 or SIRT-4 expression in the dorsal striatum^64^. METH significantly increased SIRT-4 and decreased SIRT-5 expression compared to that of the control, and piracetam exposure prevented these effects in astrocytes (Figs. 4, 5 and 6). However, the impact of cocaine, METH and morphine on mtSIRTs expression was not studied in astrocytes. The current study showed that cocaine and morphine affected the expression of mtSIRT-3, but there was no significant change in mtSIRT-5 expression (Fig. 5). The psychostimulant- and opioid-mediated excess and/or energy deficits in astrocytes may be associated with metabolic imbalance and cellular stress.

SIRT-6 binds to telomeric chromatin and deacetylates histone H3 at lysine 9. The aging-induced loss of homologous recombination (HR) is mediated via decreased SIRT-6 expression^65^. METH exposure promotes aging-dependent processes in the rat striatum by interfering with normal SIRT-6 functions^66^. Previous reports demonstrated that cocaine exposure downregulated the expression of SIRT-6 in the dorsal striatum and the medial prefrontal cortex in a rat model^67^. Cocaine, METH, and morphine caused slight decreases, but no significant changes, in SIRT-6 expression in astrocytes in the present study (Fig. 6).

SIRT-7 is the least studied mammalian sirtuin, and it plays an important role in hepatic lipid metabolism, mitochondrial homeostasis, tumorigenesis, and neurodegeneration^68,69^. SIRT-7 performs various cellular activities, including cellular proliferation, RNA polymerase I transcription, ribosome biogenesis, cell homeostasis and genome regulation^70,71^. SIRT-7-deficient mice show a reduced lifespan and develop heart hypertrophy and inflammatory cardiomyopathy^72^. Cocaine upregulated the expression of SIRT-7 in the total and nuclear fractions of astrocytes in the current study. However, morphine and METH did not cause any significant changes in SIRT-7 expression (Figs. 3, 6 and 7). A recent study demonstrated that5′ AMP-activated protein kinase (AMPK) determined the activation, subcellular distribution, and degradation of SIRT-7 in the nucleus and cytoplasm under cellular energy stress^73^. The maintenance of energy homeostasis and the execution of adaptive responses during oxidative stress are critical for cellular functions. The cellular energy sensor AMPK is activated in response to energy stress, which increases the ATP ratio to restore energy balance (Garcia and Shaw, 2017; Sivalingam et al., 2020). Previous studies noted the importance of the AMPK-mediated activation of SIRTs 1, 2, 3, 4, 6 and 7 and their interlinked functions in maintaining energy levels under various stress and disease conditions. Our recent studies demonstrated that cocaine activated AMPK expression in astrocytes and microglia^5,76,77^. These studies suggest that psychostimulants and opioid-induced energy deficit and metabolic dysfunctions may be interlinked with energy-sensing AMPKs and the modulation of SIRTs (1–7) in astrocytes.

These seven mammalian SIRTs regulate various physiological processes ranging from metabolism to epigenetic modification in many tissues^78^. Decreased levels of SIRTs and loss of deacetylation may lead to hyperacetylation and increase the expression of target genes that promote behavioral responses to psychostimulants, self-administration paradigms and neuronal impairments^78^. Because psychostimulants and opioids induced modulation of SIRT expression, increased or decreased levels play critical roles in the CNS. The systematic evaluation of new treatment medications for SUD is emerging. Effective treatments for SUDs are limited, dropout rates are high, and there is a substantial risk of relapse when treatment finishes. Therefore, we focused on investigating the protective effect of piracetam against psychostimulant drug-mediated epigenetic changes in astrocytes. Recently, new epigenetics-based perspectives and early therapeutic interventions have been developed to combat neuro-cognitive pathological effects of psychostimulant and opioids abuse^30,31^. Apart from piracetam, other studies using different neuroprotective compounds have shown protective effects against methamphetamine toxicity. Previously, Modafinil has been shown to attenuate the methamphetamine-induced neuroinflammation in in vivo study^79–81^ and protects methamphetamine-induced methylation profiles in the mouse brain^82^. A great deal of interest exists in developing sirtuin-specific activators and inhibitors as potential treatments for various diseases. Resveratrol derivatives (SRT1720, SRT2104, SRT2379) were developed to stimulate SIRT activities, and SIRT inhibitors, such as splitomicin, sirtinol, AGK2, cambinol, suramin, tenovin, salermide, and fucoidan (SIRT-1, SIRT-2, SIRT-3 and SIRT-5), were identified for the treatment of numerous disorders^46,83^.

The reversible acetylation of proteins on lysine residues is a key mechanism, which may be adjusted as circumstances demand. Sirtuin-dependent (SIRT-1, SIRT-3, SIRT-4, and SIRT-5) mitochondrial biochemical pathways perform numerous functions that are critical to cellular and organismal homeostasis. Piracetam positively regulated SIRT expression and protected astrocytes from the psychostimulant- and opioid-induced dysregulated epigenetic. These findings suggest that cocaine, METH, and morphine alter SIRT deacetylation activity, which may lead to dysregulation of nuclear and mitochondrial protein transcription. Cocaine, METH and morphine exhibited differential effects on SIRT inhibition and expression in mitochondrial and nuclear localization. It would be interesting to develop drugs that positively regulate SIRT functions and localization without the side effects of piracetam.

## Materials and methods

### Cell culture and reagents

Piracetam (purity > 99%) was purchased from Sigma-Aldrich (CAS- St. Louis, MO, USA). Cell culture reagents were purchased from ScienCell (Carlsbad, CA, USA). Primary antibodies against SIRTs (1–7) were purchased from Proteintech (Rosemont, IL, USA). Electrophoresis reagents and nitrocellulose membranes were purchased from Bio-Rad (Richmond, CA, USA). All other reagents were purchased from Sigma–Aldrich (St. Louis, MO, USA). All fluorescent secondary antibodies were purchased from Thermo Fisher Scientific (Grand Island, NY, USA).

### Primary human astrocytes and microglia

Human primary astrocytes (isolated from the cerebral cortex) were obtained from ScienCell (catalog #1800, ScienCell, Carlsbad, CA, USA). Human primary microglia (isolated from the cortex) were obtained from Celprogen (catalog #37,089–01, Torrance, CA, USA). Cultured astrocytes were maintained in astrocyte medium (catalog #1801, ScienCell, Carlsbad, CA, USA) supplemented with fetal bovine serum (FBS) at a final concentration of 2% and a 1% antibiotic/antimycotic solution (ScienCell, Carlsbad, CA, USA). Cultured microglia were maintained in complete human microglia primary tissue culture growth media with antibiotics (cat #M37089-0, Celprogen, Torrance, CA) supplemented with FBS.

### Drug treatment

Piracetam, cocaine, methamphetamine and morphine were prepared in cell culture-grade distilled water to obtain working concentrations. To investigate the protective effects of piracetam, cultured cells were divided into eight groups. Briefly, primary human astrocytes (2 × 10^6^) were exposed to the following conditions for 24 h: (i) medium alone in control cells, (ii) piracetam (10 µM), (iii) cocaine (1 µM), (iv) piracetam and cocaine, (v) METH (10 µM), (vi) piracetam and METH, (vii) morphine (5 µM), and (viii) piracetam in combination with morphine. human primary microglia. Microglia (2 × 10^6^) were exposed to METH (5 µM), cocaine (1 µM), and morphine (1 µM) for 24 h. The optimized doses and time responses used in the present studies were based on our published report^33,84,85^. At the end of the incubation period, cell pellets were extracted, and the differential expression of SIRTs gene and protein expression, modifications and intracellular, mitochondrial and nuclear localization were examined using confocal microscopy.

### RNA extraction and real-time quantitative PCR (qRT-PCR)

Total RNA from human primary astrocytes was extracted using the Qiagen Kit (Invitrogen Life Technologies, Carlsbad, CA, USA) according to the manufacturer’s instructions. For RNA extraction and real-time quantitative PCR, we followed the procedure according to our previous study^86^. The purity of the extracted RNA was determined by measuring absorbance in a NanoDrop (Thermo Scientific, Waltham, MA, USA). First-strand cDNA was synthesized from total RNA (1000 ng) samples. cDNA amplification was performed using specific primers for SIRT-1 (Hs01009006_m1), SIRT-2 (Hs00247263_m1), SIRT-4 (Hs01015516_g1), SIRT-7 (Hs01034735_m1) and β-actin (Hs99999903_m1) (Applied Biosystems, Foster City, CA). The β-actin gene was used as an endogenous control for quantification of real-time PCR amplification under different experimental conditions. The following PCR protocol was used: initial denaturation at 95 °C for 2 min, followed by 40 cycles of cyclic denaturation at 95 °C for 3 s and annealing at 60 °C for 30 s.

Relative mRNA expression was quantitated, and the mean fold-change in expression of the target gene was calculated using the comparative ΔΔCT method (transcript accumulation index, TAI = 2−^ΔΔCT^). The results of RNA from treated samples were normalized to results obtained from the control (untreated) sample. Three independent experiments were performed in triplicate to ensure reproducibility.

### Western blot analyses of total, mitochondrial and nuclear fractions

SIRT (1–7) protein modifications were analyzed using Western blotting.

#### Total cell lysate

Astrocytes were grown and exposed to cocaine, METH and morphine alone or in combination with piracetam for 24 h. After the incubation period, cells were collected, washed twice in 1X PBS, resuspended in lysis buffer (Thermo Scientific, USA) for 30 min, and centrifuged for 15 min at 13,000 × g at 4 °C to collect the total lysates for Western blot analyses according to the manufacturer’s protocol.

#### Isolation of mitochondrial fraction

The Mitochondrial Isolation Kit (Abcam, CA) was used to isolate mitochondria from cocaine-, METH- and morphine- and/or piracetam-treated cells. Briefly, primary astrocytes (2.5 × 10^8^) were grown and exposed to cocaine, METH and morphine and/or piracetam for 24 h. At the end of the experimental period, the cells were trypsinized and washed with PBS. The collected cells were centrifuged (1000 × g for 10 min) at room temperature (RT), and the pellet was suspended in mitochondrial assay buffer. After 2 min of incubation on ice, the cells were homogenized with a glass homogenizer using 20 up-down strokes. According to the manufacturer’s protocol, the supernatant was centrifuged at 5,000 × g for 10 min at 4 °C to collect the final mitochondrial fraction. For isolation of mitochondrial fraction, we followed the procedure according to our previous study^75^. The pellet was resuspended in lysis buffer to examine the mitochondrial protein expression levels.

#### Isolation of the nuclear fraction

The NE-PER Nuclear Isolation Kit (Thermo Scientific, USA) was used to extract the nuclear fractions from different experimental groups. For isolation of nuclear fraction, we followed the procedure according to our previous study^75^. Briefly, primary astrocytes (2.5 × 10^8^) were grown and exposed to cocaine, METH and morphine and/or piracetam for 24 h. After the incubation time, cells were trypsinized and washed with PBS. The collected cells were centrifuged (500 × g for 5 min), and the pellets were suspended in different nuclear extraction assay buffers according to the manufacturer’s protocol. The extracted nuclear fraction was used to examine the nuclear protein expression levels.

Equal amounts of total, mitochondrial and nuclear proteins were resolved using 4–15% gradient polyacrylamide gel electrophoresis, and separated proteins were subsequently transferred to a nitrocellulose membrane. The membranes were blocked with 5% nonfat milk in TBST at RT for 1 h and incubated at 4 °C overnight with antibodies against SIRT-1 (13,161–1-AP), SIRT-2 (19,655–1-AP), SIRT-3 (10,099–1-AP), SIRT-4 (66,543–1-Ig), SIRT-5 (15,122–1-AP), SIRT-6 (E-AB-14344), SIRT-7 (12,994–1-AP), lamin (10,298–1-AP), COX-IV (11,242–1-AP) and GAPDH (10,494–1-AP) prepared in TBST (1:1000). After washing with TBST, the blots were incubated with peroxidase-conjugated secondary antibodies (EpiGentek, NY, USA) for 1 h. Immunoreactive bands were visualized using a chemiluminescence reagent. To ensure equal protein loading, COX-IV, lamin and GAPDH were used as internal controls. Densitometric analyses were performed using ImageJ digitalizing software.

### Intracellular ATP analysis

Total cellular ATP was measured in astrocytes using a luminescent ATP detection assay kit (Abcam, ab113849, USA) according to the manufacturer’s protocol. Briefly, astrocytes (5 × 10^4^ cells/well) were grown in 96-well plates and exposed to cocaine, METH morphine and/or piracetam for 24 h. After the experimental period, cells were lysed and exposed to an ATP substrate solution for measurement of luminescence intensity in a luminometer. The concentration of ATP was proportional to the measured luminescence counts.

### Immunofluorescence staining

Briefly, astrocytes (2 × 10^4^ cells/mL) were grown on multichambered slides and exposed to cocaine, METH and morphine and/or piracetam for 24 h. At the end of the incubation period, the cells were fixed with 4% paraformaldehyde for 15–30 min at room temperature followed by permeabilization with 0.2% Triton X-100 in PBS for 15 min at RT. For immunofluorescence staining, we followed the procedure according to our previous study^86^. The cells were blocked with 5% normal goat serum for 1 h at RT. SIRT-1 (13,161–1-AP), SIRT-2 (19,655–1-AP), SIRT-3 (10,099–1-AP), SIRT-7 (12,994–1-AP), and COX-IV (11,242–1-AP) antibodies were used for immunofluorescence. The appropriate primary antibody (diluted 1: 300) was subsequently added, and the slides were incubated overnight at 4 °C. After washing with PBS, the cells were incubated with specific secondary antibodies (Thermo Fisher Scientific, USA). Cellular nuclei were stained with 4′6-diamidino-2-phenylindole (DAPI, Vector Laboratories, USA). Slides were examined with a C1-plus Nikon laser-scanning confocal microscope. The fluorescence intensity (calculated as the corrected total cell fluorescence (CTCF)) was measured using ImageJ software. We performed colocalization analysis using the Plug-In, and COLOC 2 algorithms of ImageJ to measure the Pearson’s coefficient r-value in three different regions of interest (ROIs) for the same cell.

### Statistical analysis

Statistical analyses were performed using GraphPad Prism version 6. Differences between the control, cocaine alone, METH alone and morphine alone were calculated using one-way ANOVA. For data in which more than two groups were compared, two-way ANOVA followed by Tukey’s post hoc test was performed. The comparisons between control Vs psychostimulants and control Vs opioid were calculated. Moreover, cocaine Vs Cocaine + piracetam, METH Vs METH + Piracetam and Morphine Vs Morphine + Piracetam comparisons were calculated using two-way ANOVA test. All data are presented as means ± SD and considered statistically significant when p < 0.05.

## Supplementary Information

Supplementary Information 1.

Supplementary Information 2.

Supplementary Information 3.

Supplementary Information 4.

Supplementary Information 5.

Supplementary Information 6.
