# Supplementary material for: Influence of psychostimulants and opioids on epigenetic modification of class III histone deacetylase (HDAC)-sirtuins in glial cells
Source: Sci Rep. 2021 Oct 29;11:21335. doi: 10.1038/s41598-021-00836-z (PMC8556237; doi:10.1038/s41598-021-00836-z)
Supplement: Supplementary file 2 — Supplementary Information 2. [file 41598_2021_836_MOESM2_ESM.pdf]

Figure 2

A

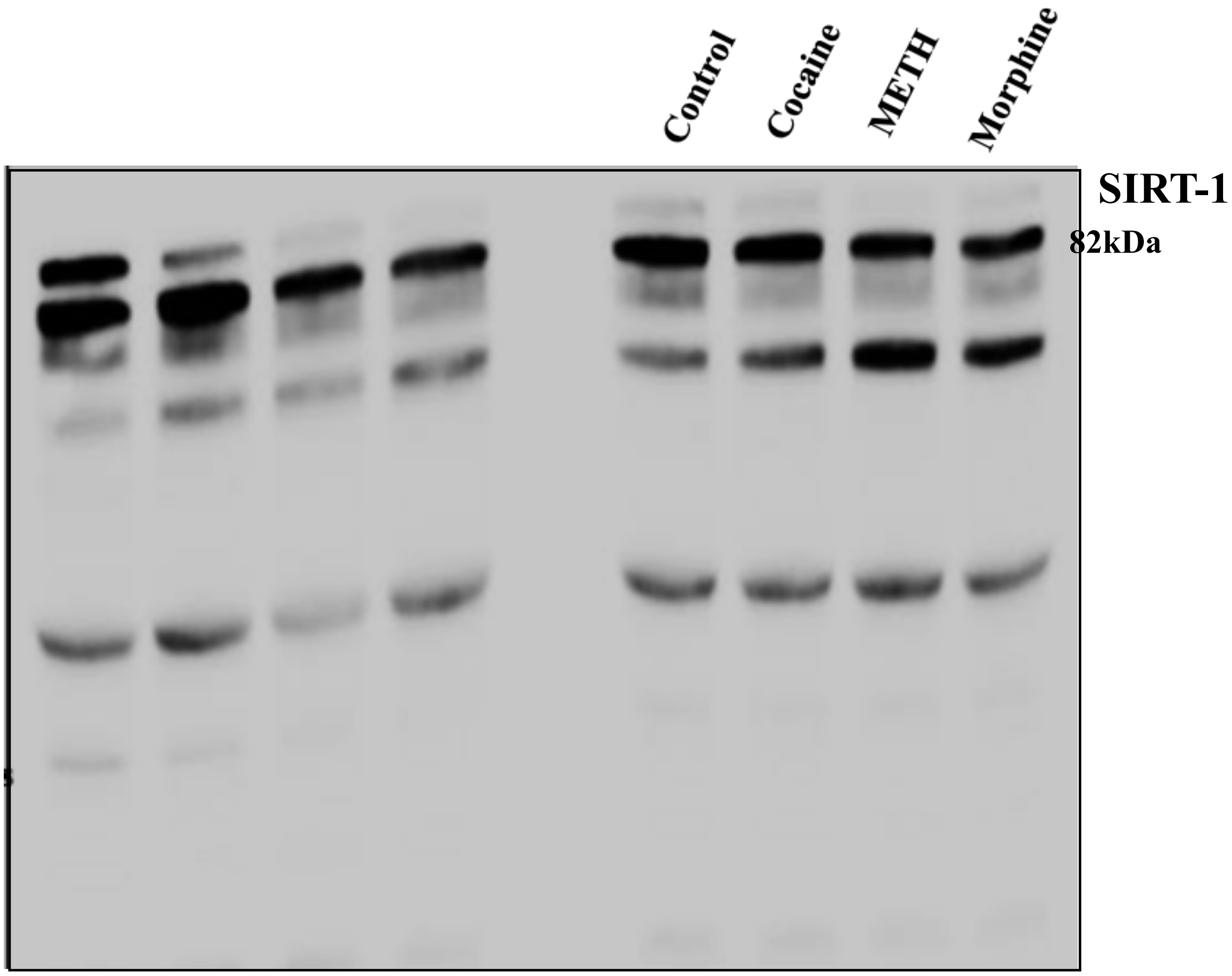

Figure 2 A: Effects of psychostimulants and opioids on SIRT-1 in human primary microglia.
The representative blot shows SIRT-1 protein level in control, cocaine (1 µM), METH (10 µM) and morphine (5 µM).

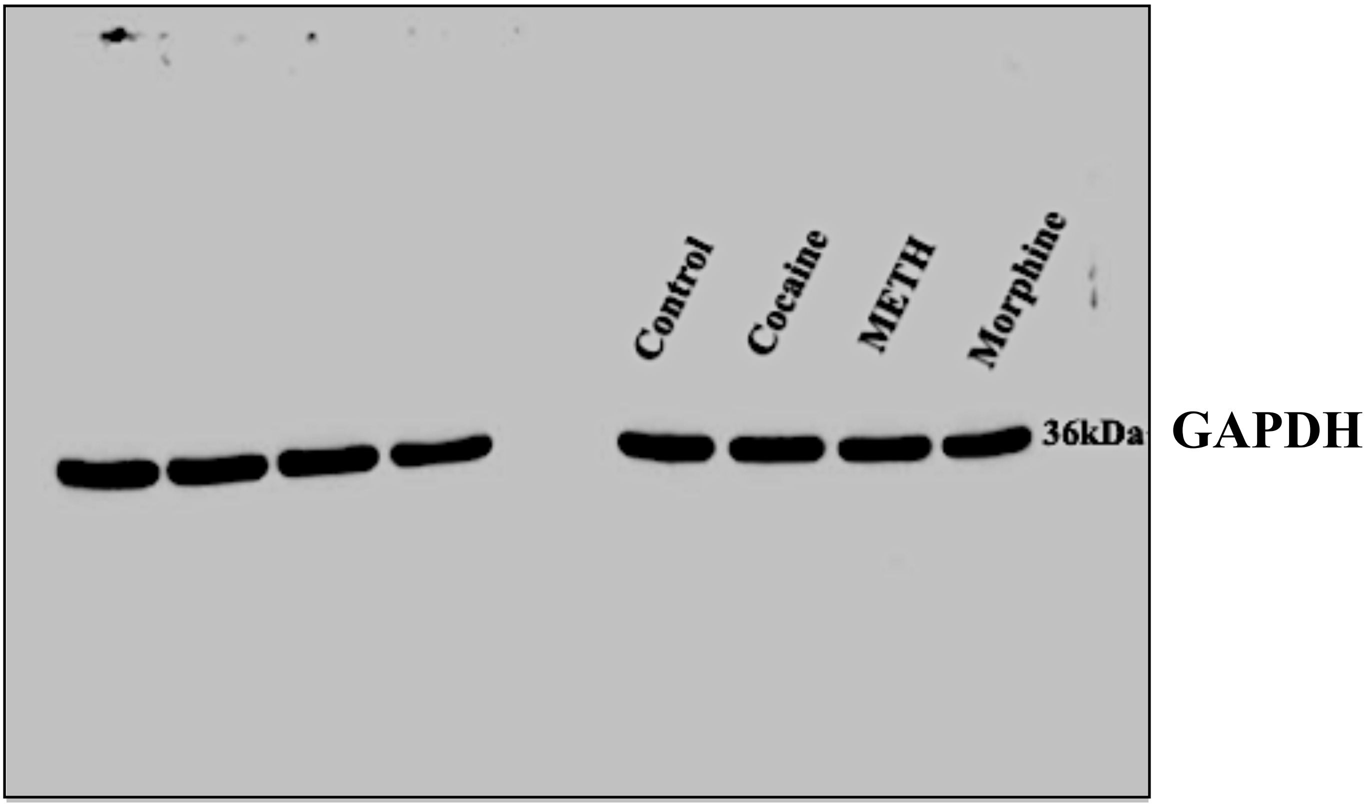

Figure 2 A: GAPDH for SIRT-1 in human primary microglia.
The representative blot shows GAPDH for SIRT-1 in control, cocaine (1 µM), METH (10 µM) and morphine (5 µM).

Figure 2

B

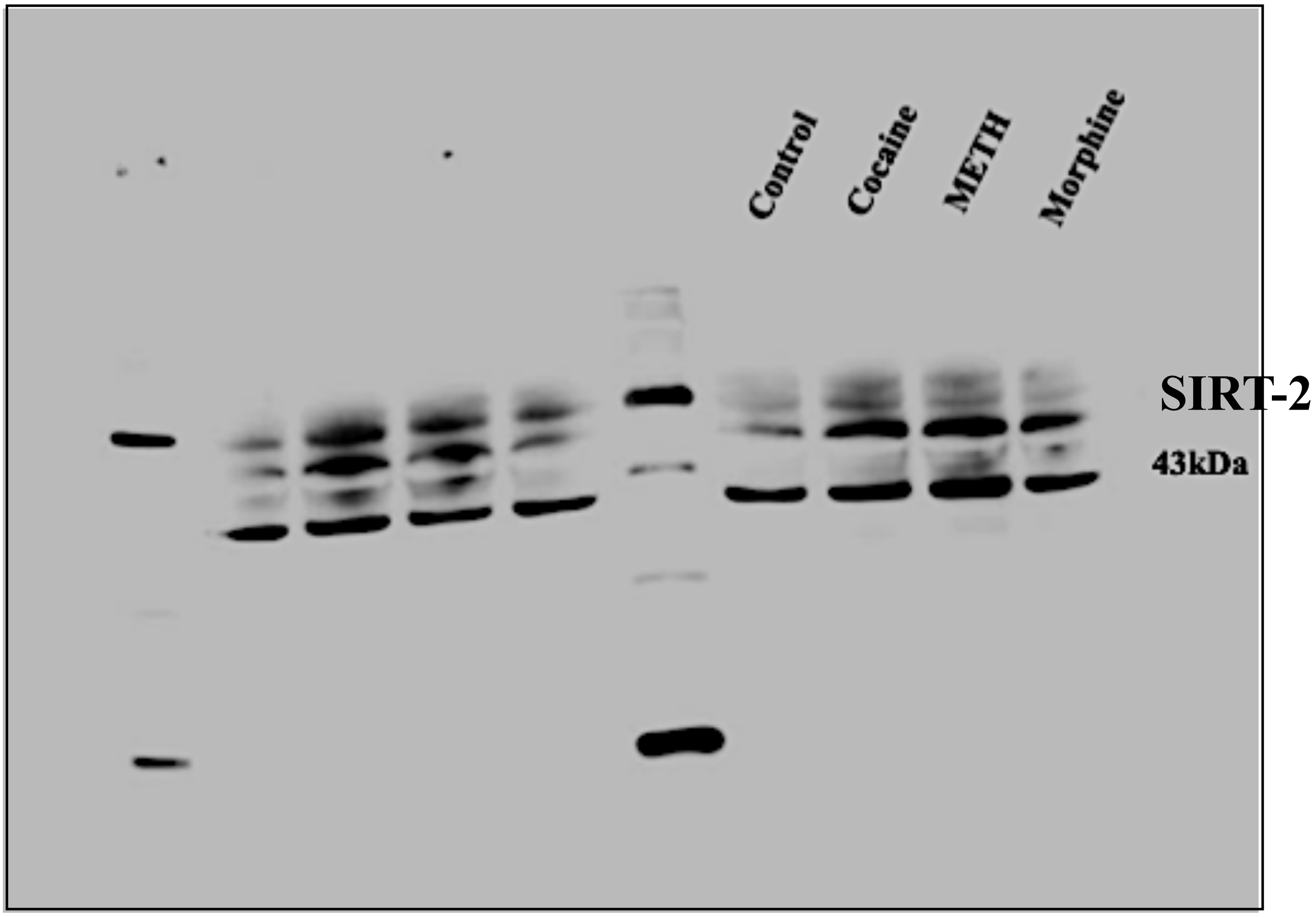

Figure 2 B: Effects of psychostimulants and opioids on SIRT-2 in human primary microglia.
The representative blot shows SIRT-2 protein level in control, cocaine (1 µM), METH (10 µM) and morphine (5 µM).

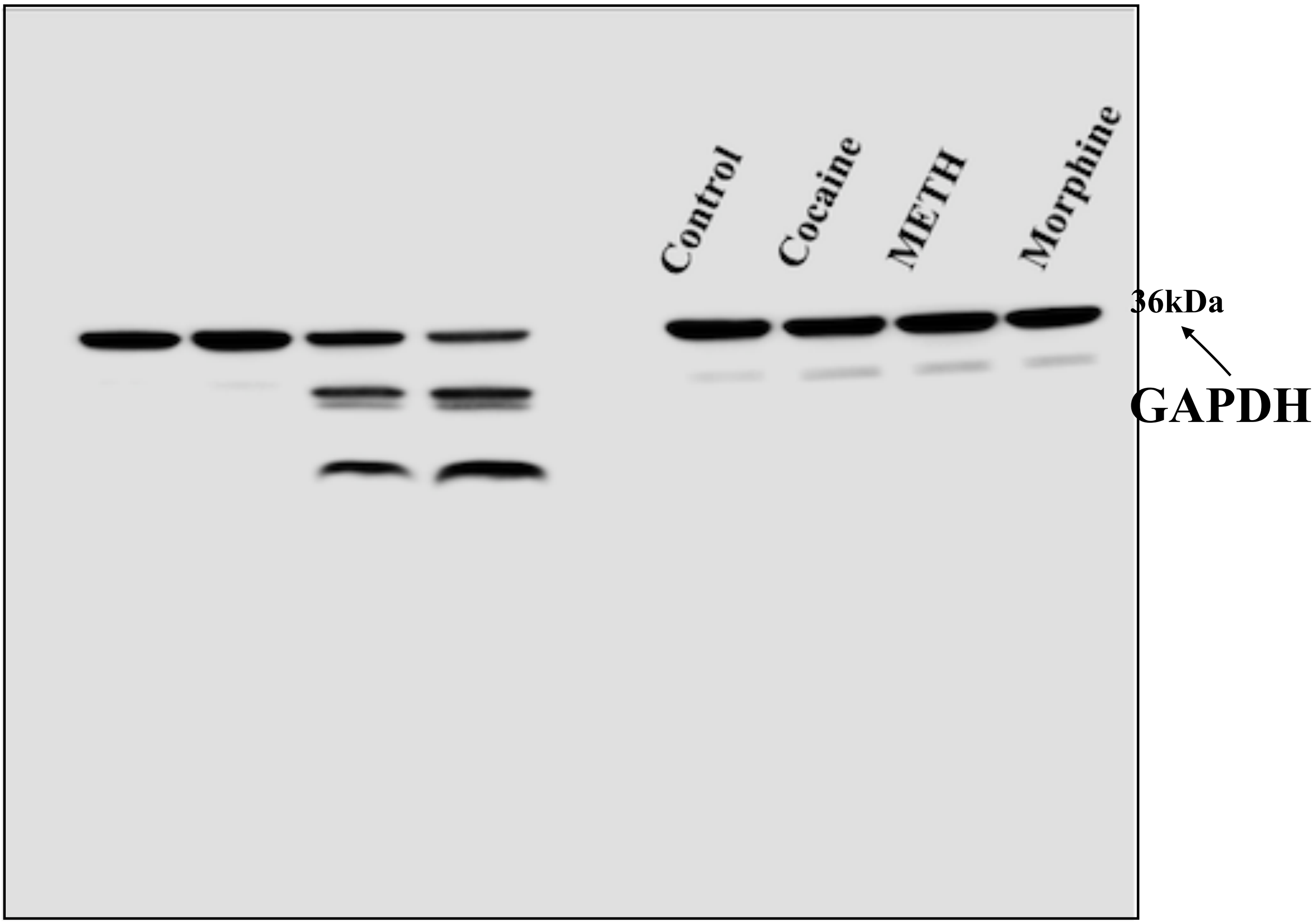

Figure 2 B: GAPDH for SIRT-2 in human primary microglia.
The representative blot shows GAPDH for SIRT-2 in control, cocaine (1 µM), METH (10 µM) and morphine (5 µM).

Figure 2

C

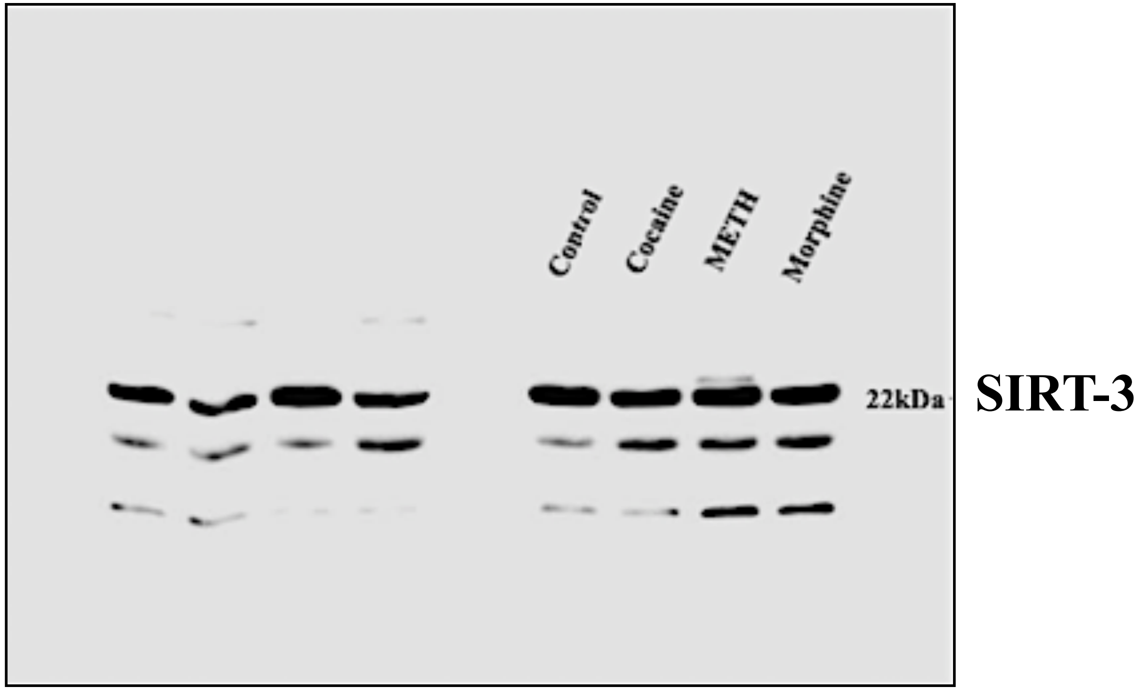

Figure 2 C: Effects of psychostimulants and opioids on SIRT-3 in human primary microglia.
The representative blot shows SIRT-3 protein level in control, cocaine (1 µM), METH (10 µM) and morphine (5 µM).

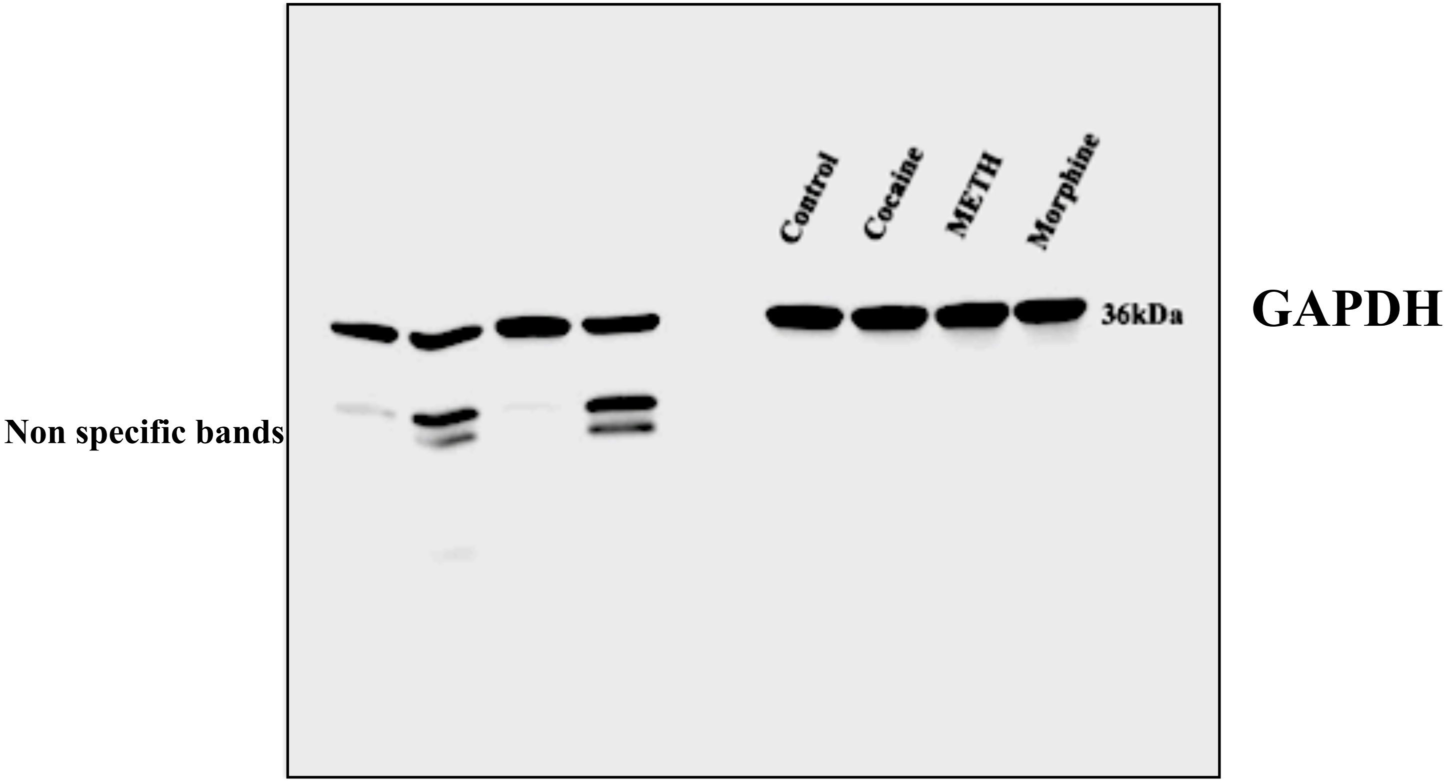

Figure 2 C: GAPDH for SIRT-3 in human primary microglia.
The representative blot shows GAPDH for SIRT-3 in control, cocaine (1 µM), METH (10 µM) and morphine (5 µM).

Figure 2

G

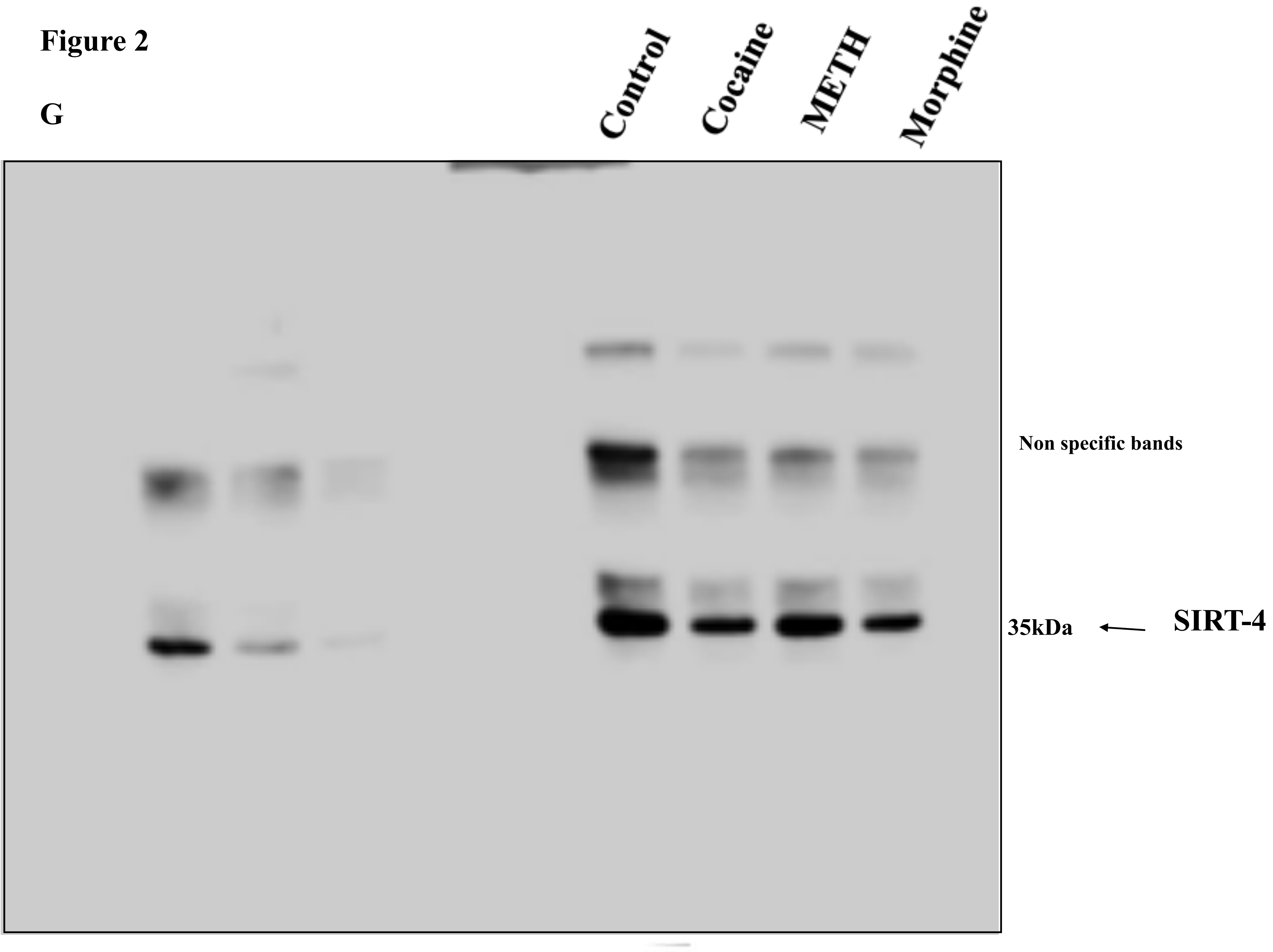

Figure 2 G: Effects of psychostimulants and opioids on SIRT-4 in human primary microglia. The representative blot shows SIRT-4 protein level in control, cocaine (1 µM), METH (10 µM) and morphine (5 µM).

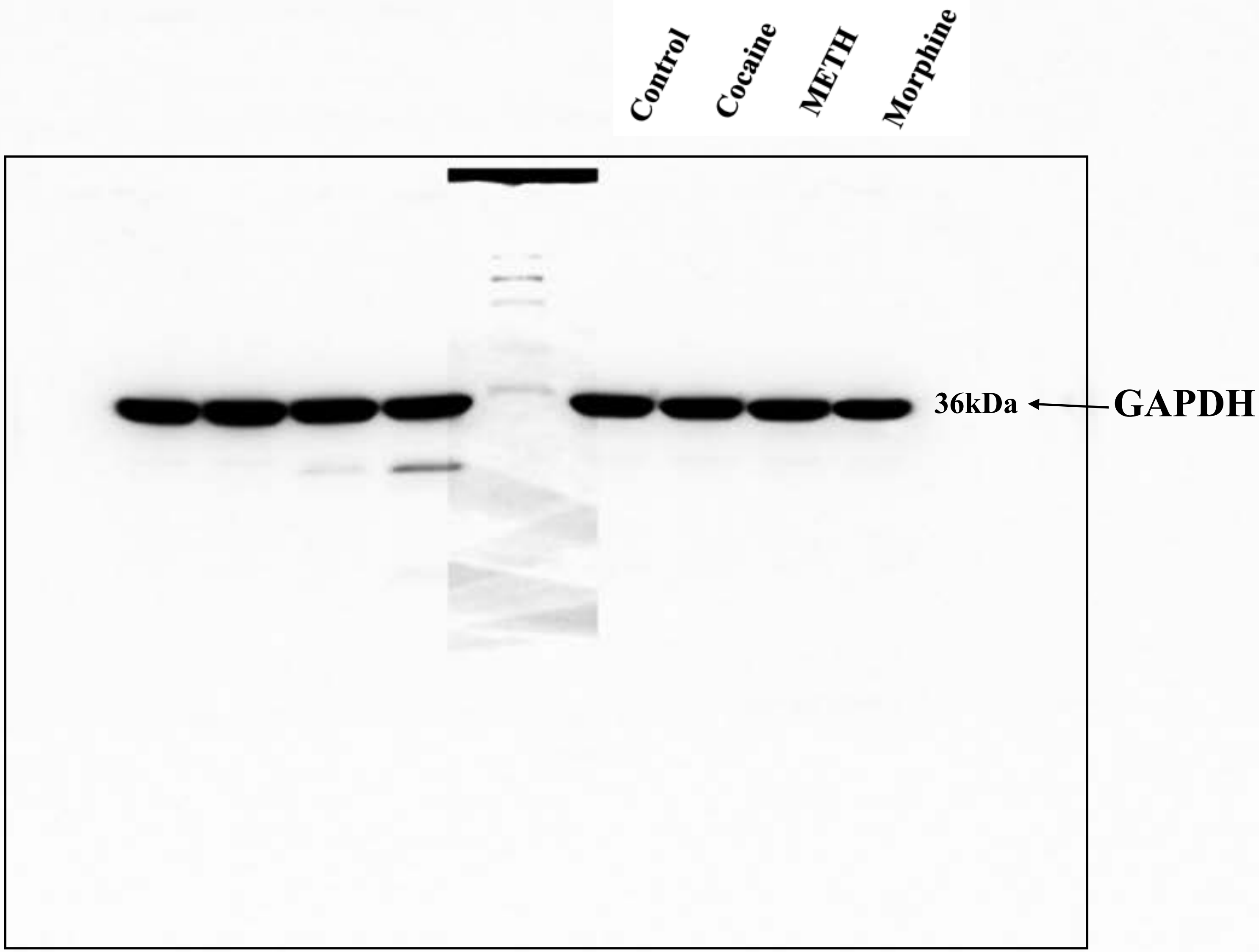

Figure 2 G: GAPDH for SIRT-4 in human primary microglia.
The representative blot shows GAPDH for SIRT-4 in control, cocaine (1 µM), METH (10 µM) and morphine (5 µM).

Figure 2

H

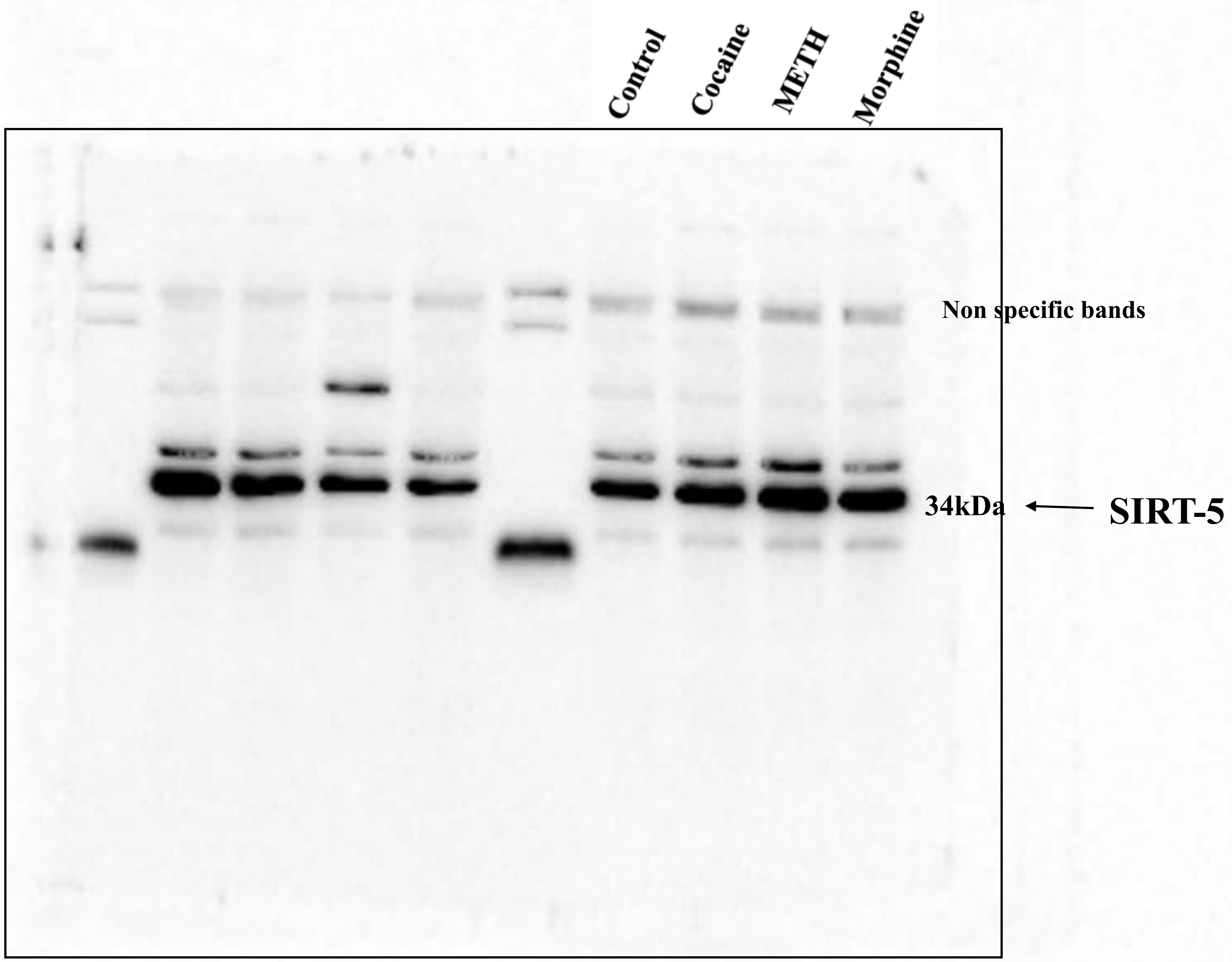

Figure 2 H: Effects of psychostimulants and opioids on SIRT-5 in human primary microglia. **The representative blot shows SIRT-5 protein level in control, cocaine (1 µM), METH (10 µM) and morphine (5 µM).**

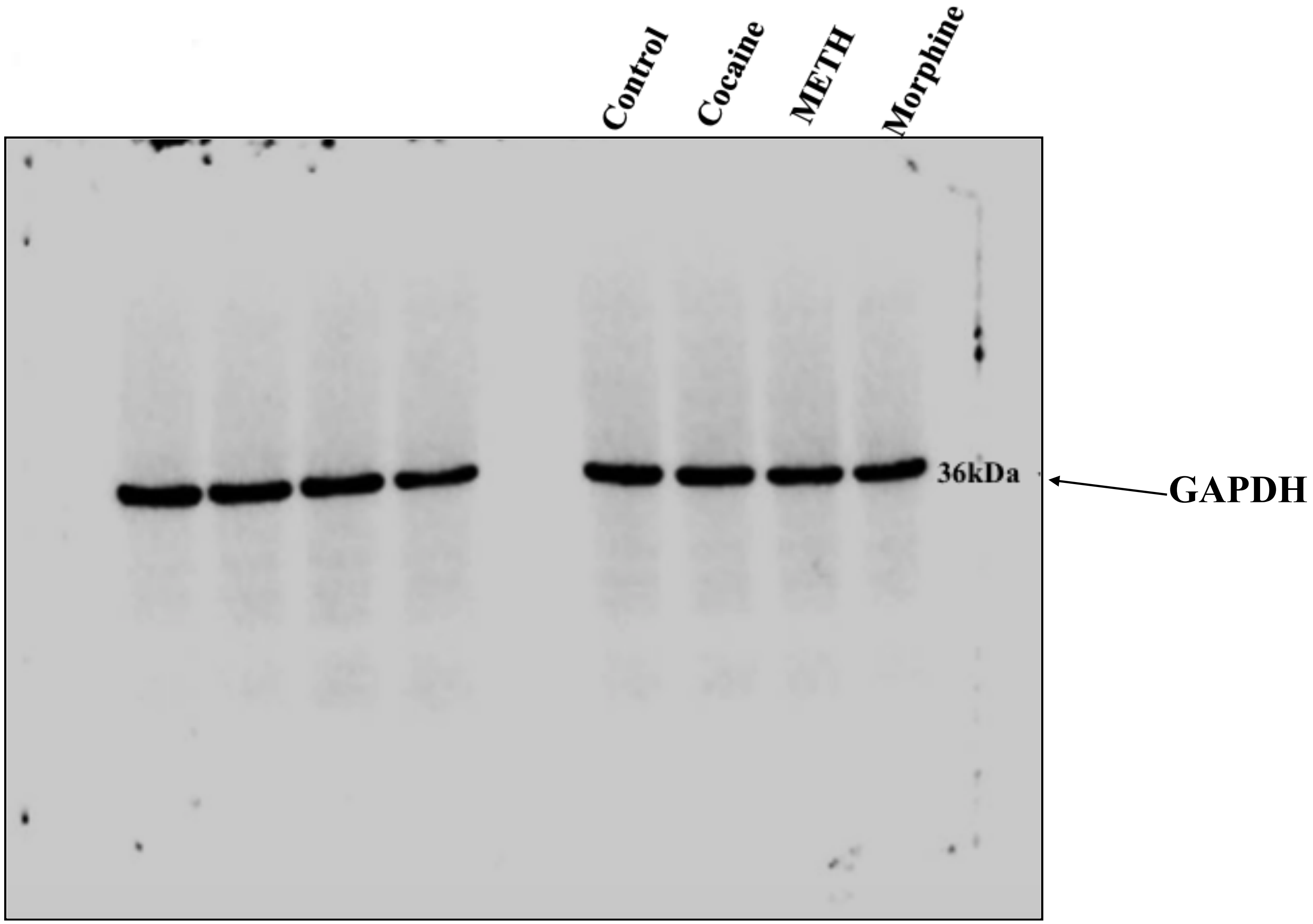

Figure 2 H: GAPDH for SIRT-5 in human primary microglia.
The representative blot shows GAPDH for SIRT-5 in control, cocaine (1 µM), METH (10 µM) and morphine (5 µM).

Figure 2

K

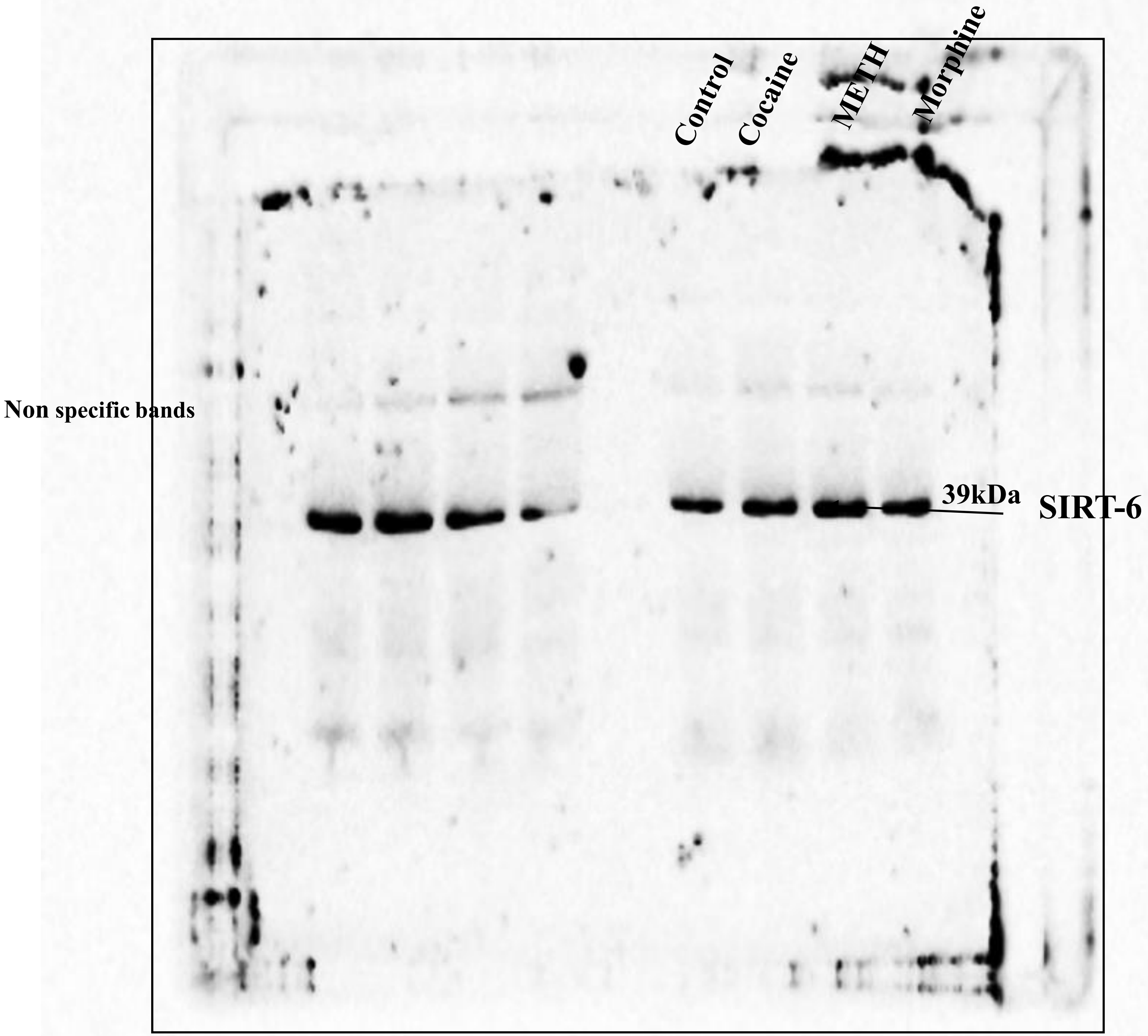

Figure 2 K: Effects of psychostimulants and opioids on SIRT-6 in human primary microglia.
The representative blot shows SIRT-6 protein level in control, cocaine (1 µM), METH (10 µM) and morphine (5 µM).

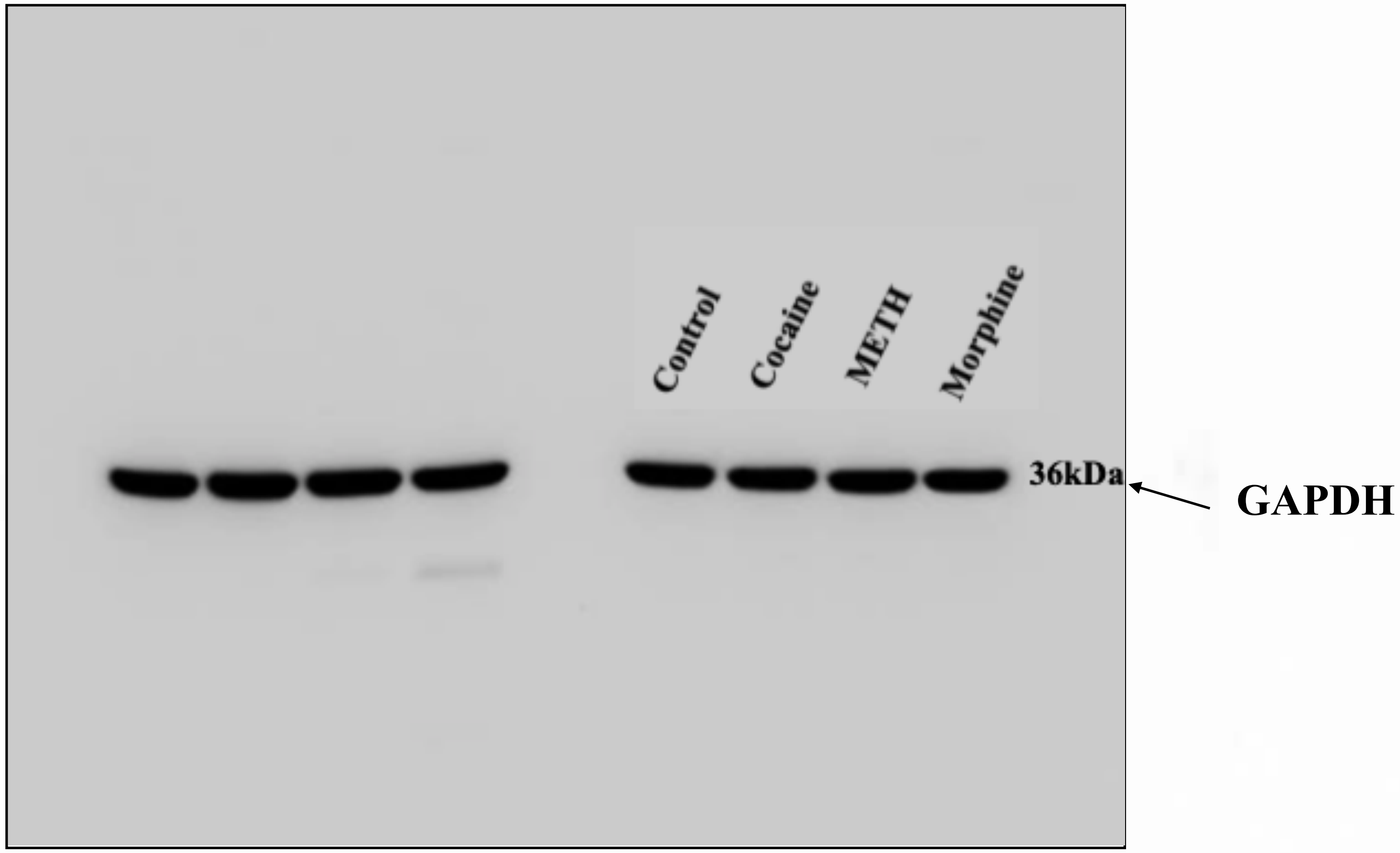

Figure 2 K: GAPDH for SIRT-6 in human primary microglia.
The representative blot shows GAPDH for SIRT-6 in control, cocaine (1 µM), METH (10 µM) and morphine (5 µM).

Figure 2

L

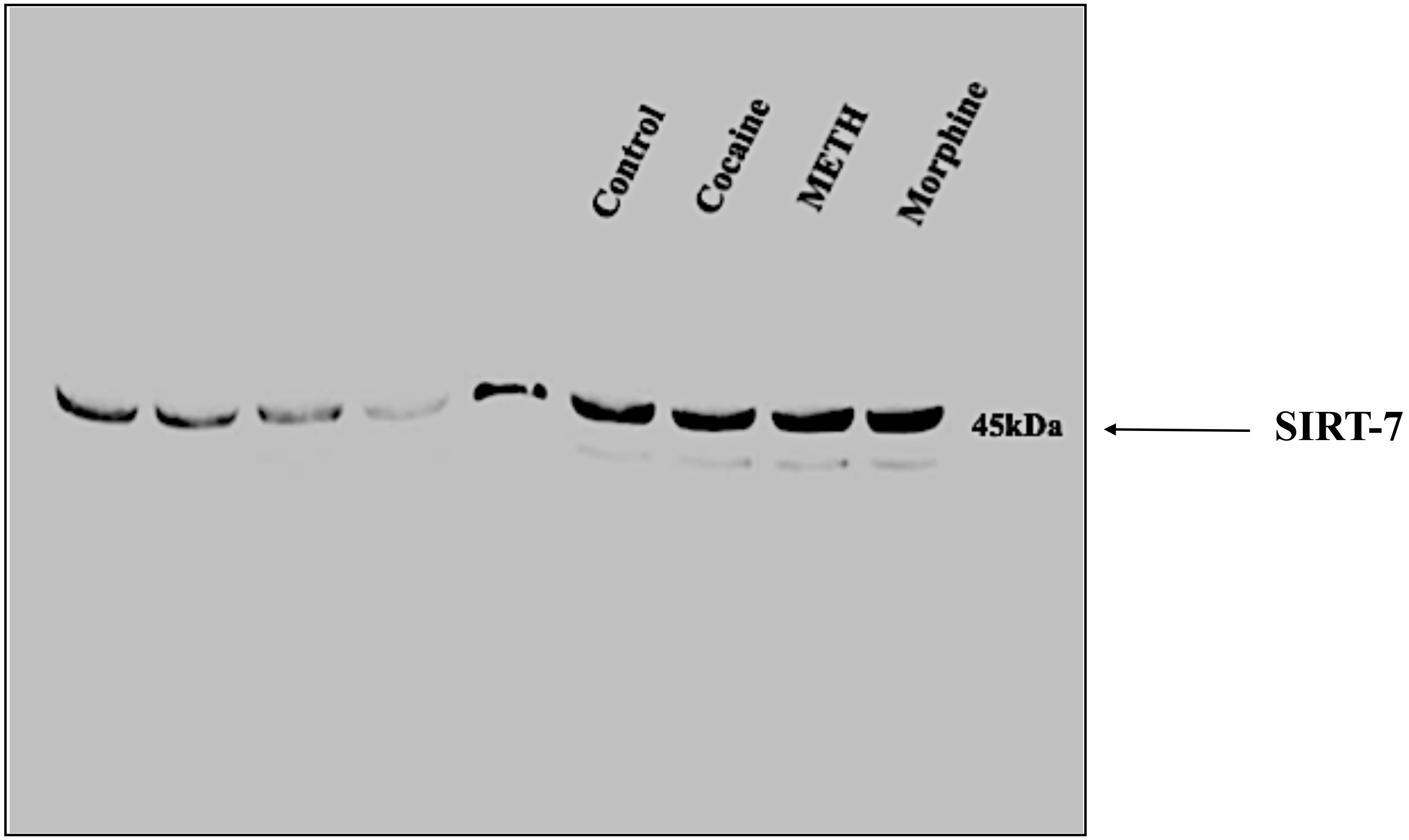

Figure 2 L: Effects of psychostimulants and opioids on SIRT-7 in human primary microglia.
The representative blot shows SIRT-7 protein level in control, cocaine (1 µM), METH (10 µM) and morphine (5 µM).

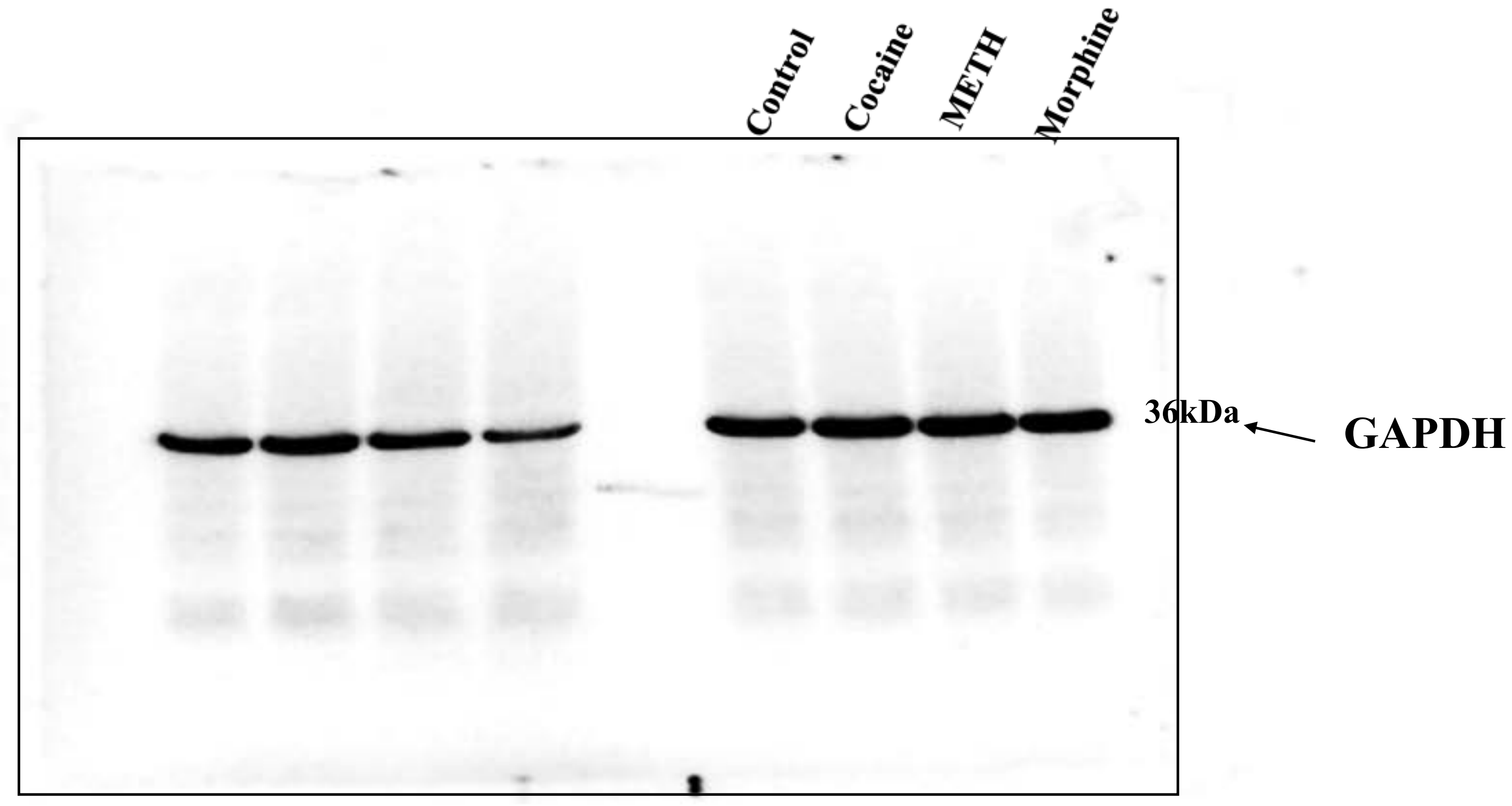

Figure 2 L: GAPDH for SIRT-7 in human primary microglia.
The representative blot shows GAPDH for SIRT-7 in control, cocaine (1 μM), METH (10 μM) and morphine (5 μM).
