# Supplementary material for: Influence of psychostimulants and opioids on epigenetic modification of class III histone deacetylase (HDAC)-sirtuins in glial cells
Source: Sci Rep. 2021 Oct 29;11:21335. doi: 10.1038/s41598-021-00836-z (PMC8556237; doi:10.1038/s41598-021-00836-z)
Supplement: Supplementary file 3 — Supplementary Information 3. [file 41598_2021_836_MOESM3_ESM.pdf]

Figure 4

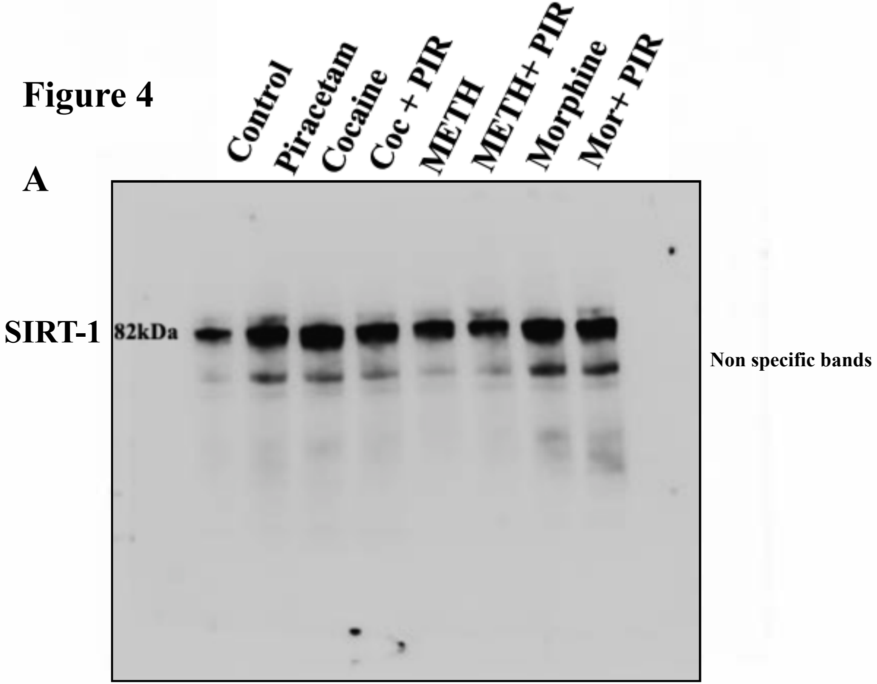

Figure 4 A: Protective Effect of piracetam against psychostimulants and opioids on SIRT-1 in human primary astrocytes.

The representative blot shows SIRT-1 protein level in control, cocaine (1 µM), METH (10 µM) and morphine (5 µM) alone or in combination with piracetam (10 µM)

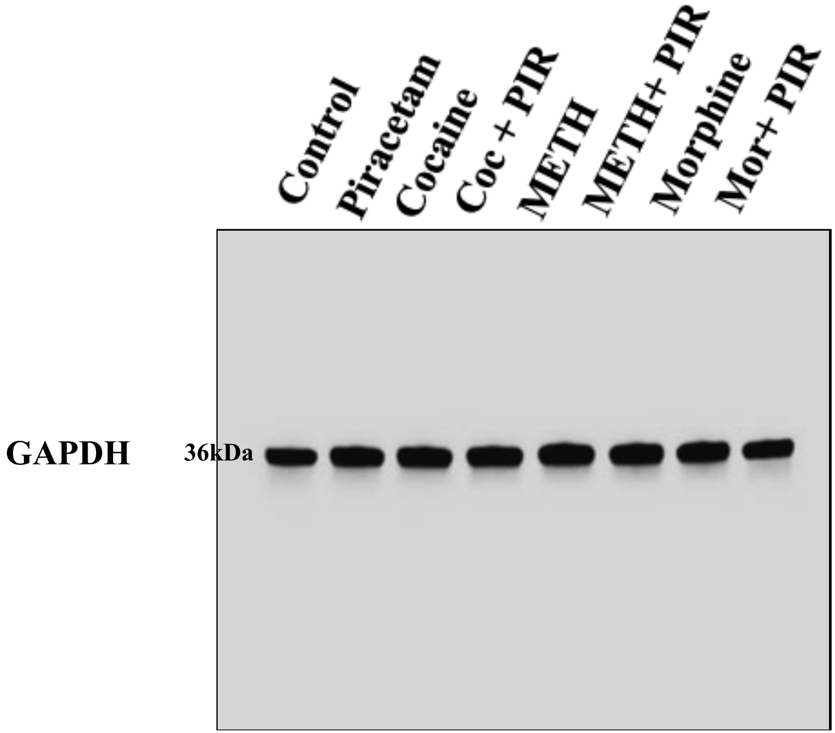

Figure 4 A: GAPDH for SIRT-1 in human primary astrocytes.
The representative blot shows GAPDH for SIRT-1 in control, cocaine (1 μM), METH (10 μM) and morphine (5 μM) alone or in combination with piracetam (10 μM)

Figure 4

B

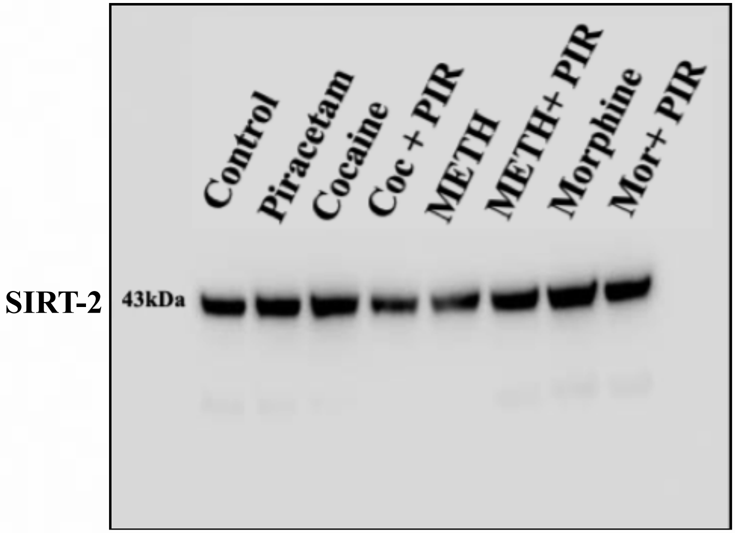

Figure 4 B: Protective Effect of piracetam against psychostimulants and opioids on SIRT-2 in human primary astrocytes.
The representative blot shows SIRT-2 protein level in control, cocaine (1 µM), METH (10 µM) and morphine (5 µM) alone or in combination with piracetam (10 µM)

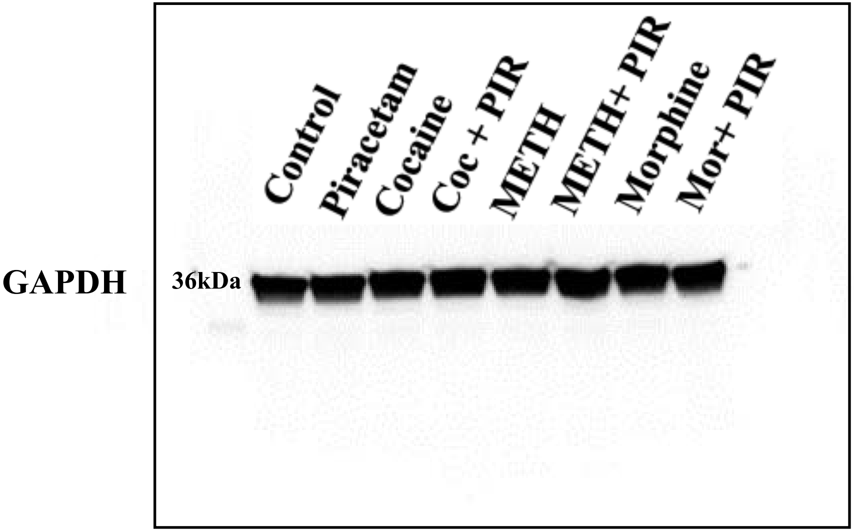

Figure 4 B: GAPDH for SIRT-2 in human primary astrocytes.
The representative blot shows GAPDH for SIRT-2 in control, cocaine (1 µM), METH (10 µM) and morphine (5 µM) alone or in combination with piracetam (10 µM)

Figure 4

C

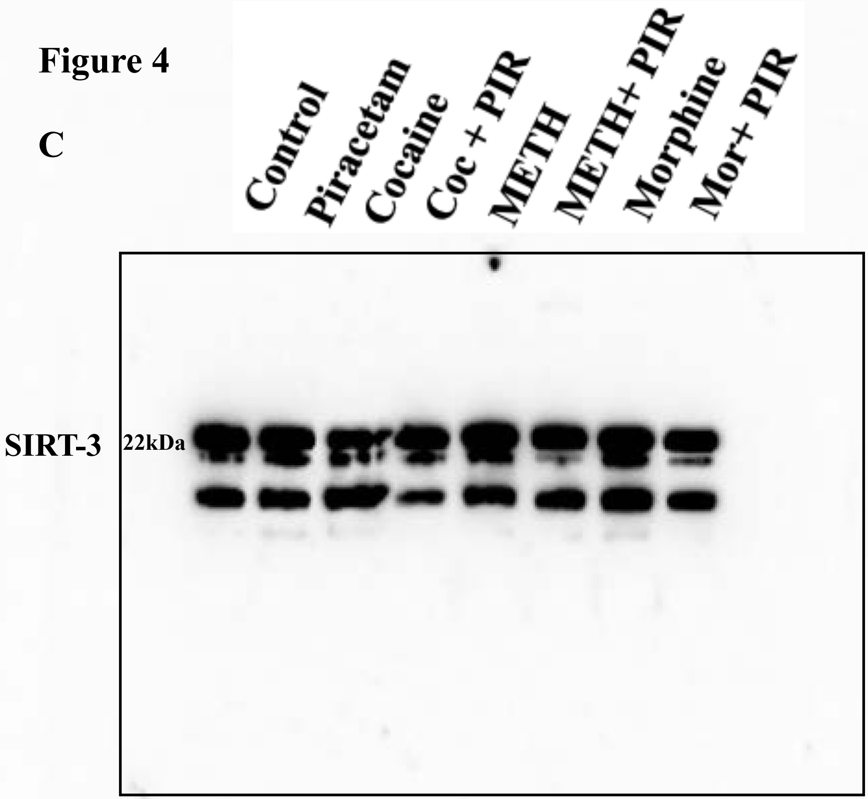

Figure 4 C: Protective Effect of piracetam against psychostimulants and opioids on SIRT-3 in human primary astrocytes.
The representative blot shows SIRT-3 protein level in control, cocaine (1 µM), METH (10 µM) and morphine (5 µM) alone or in combination with piracetam (10 µM)

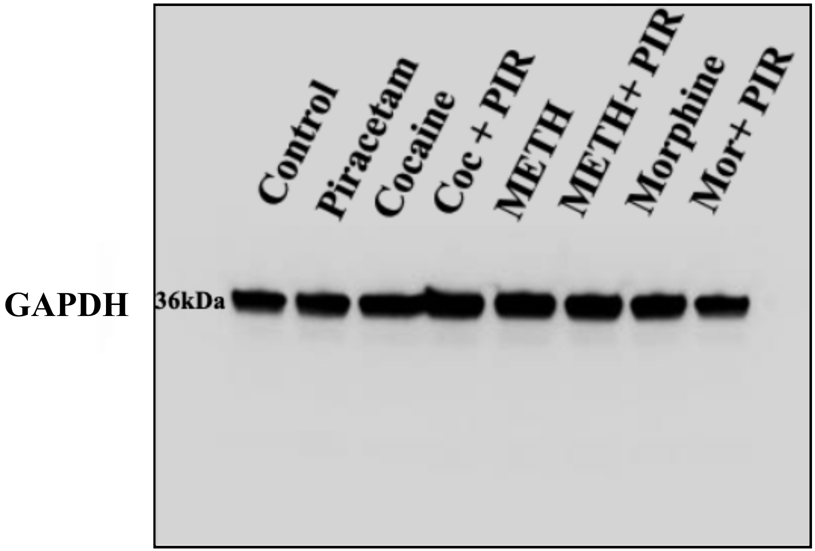

Figure 4 C: GAPDH for SIRT-3 in human primary astrocytes.
The representative blot shows GAPDH for SIRT-3 in control, cocaine (1 µM), METH (10 µM) and morphine (5 µM) alone or in combination with piracetam (10 µM)
