# Supplementary material for: Influence of psychostimulants and opioids on epigenetic modification of class III histone deacetylase (HDAC)-sirtuins in glial cells
Source: Sci Rep. 2021 Oct 29;11:21335. doi: 10.1038/s41598-021-00836-z (PMC8556237; doi:10.1038/s41598-021-00836-z)
Supplement: Supplementary file 4 — Supplementary Information 4. [file 41598_2021_836_MOESM4_ESM.pdf]

Figure 5

A

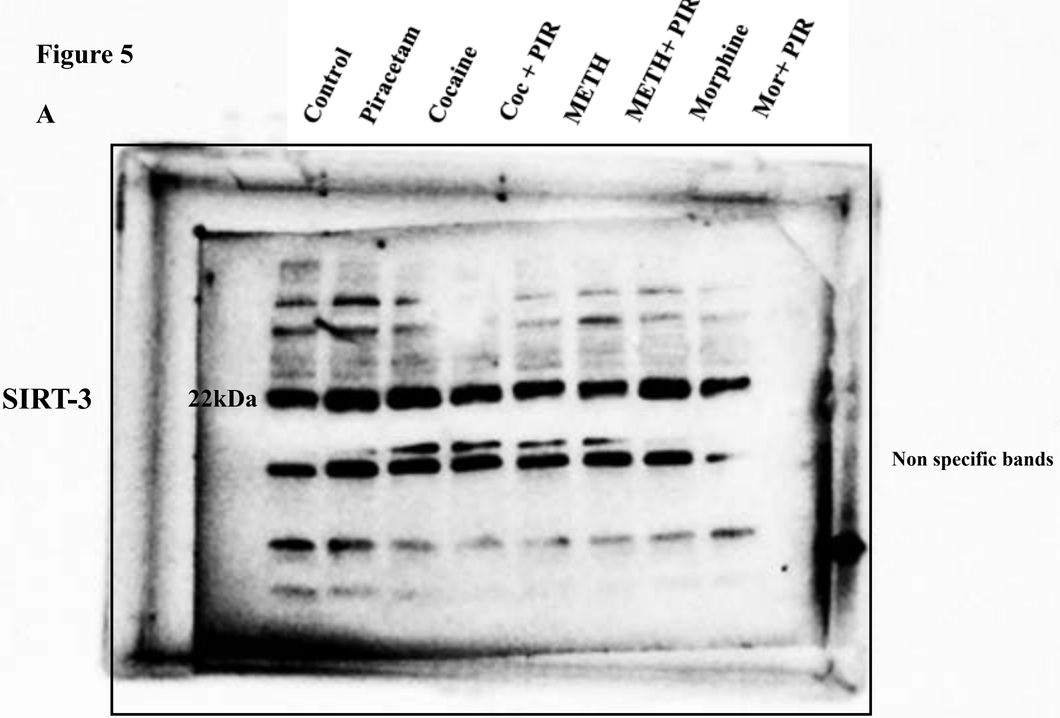

Figure 5 A: Protective Effect of piracetam against psychostimulants and opioids on SIRT-3 in human primary astrocytes.

The representative blot shows SIRT-3 protein level in control, cocaine (1 µM), METH (10 µM) and morphine (5 µM) alone or in combination with piracetam (10 µM)

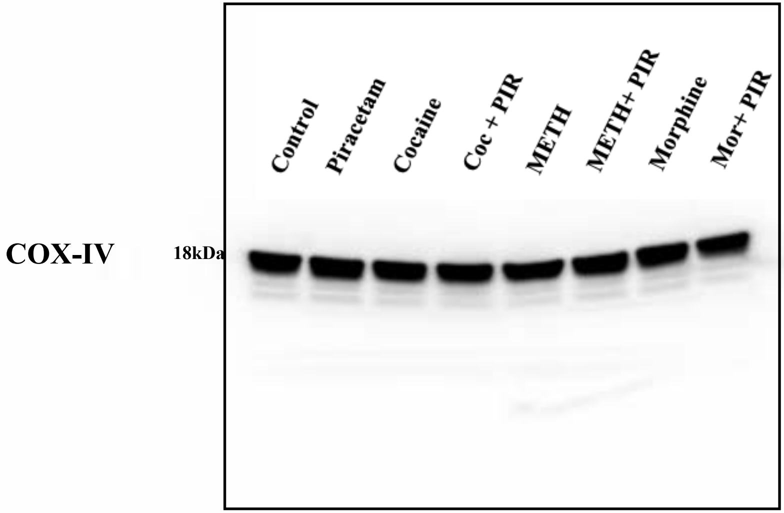

Figure 5 A: COX-IV for SIRT-3 in human primary astrocytes.
The representative blot shows COX-IV for SIRT-3 in control, cocaine (1 µM), METH (10 µM) and morphine (5 µM) alone or in combination with piracetam (10 µM)

Figure 5

C

SIRT-5

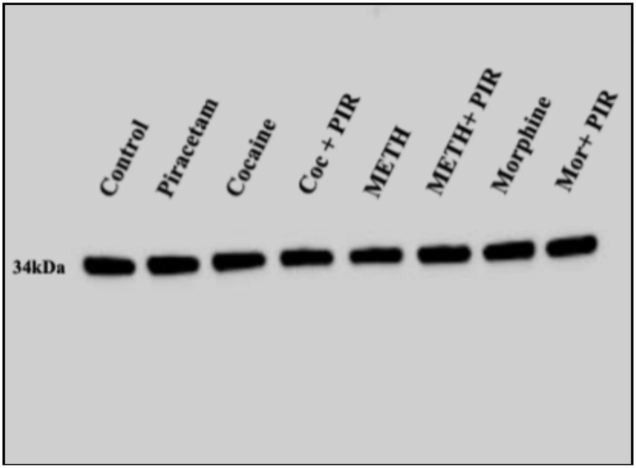

Figure 5 C: Protective Effect of piracetam against psychostimulants and opioids on SIRT-5 in human primary astrocytes.
The representative blot shows SIRT-5 protein level in control, cocaine (1 µM), METH (10 µM) and morphine (5 µM) alone or in combination with piracetam (10 µM)

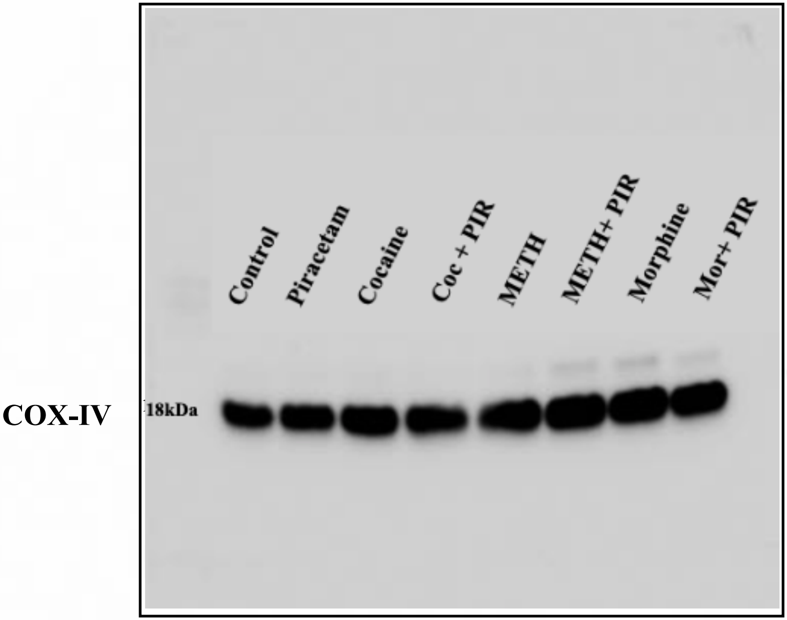

Figure 5 C: COX-IV for SIRT-5 in human primary astrocytes.
The representative blot shows COX-IV for SIRT-5 in control, cocaine (1 µM), METH (10 µM) and morphine (5 µM) alone or in combination with piracetam (10 µM)
