# Supplementary material for: Influence of psychostimulants and opioids on epigenetic modification of class III histone deacetylase (HDAC)-sirtuins in glial cells
Source: Sci Rep. 2021 Oct 29;11:21335. doi: 10.1038/s41598-021-00836-z (PMC8556237; doi:10.1038/s41598-021-00836-z)
Supplement: Supplementary file 5 — Supplementary Information 5. [file 41598_2021_836_MOESM5_ESM.pdf]

Figure 6

A

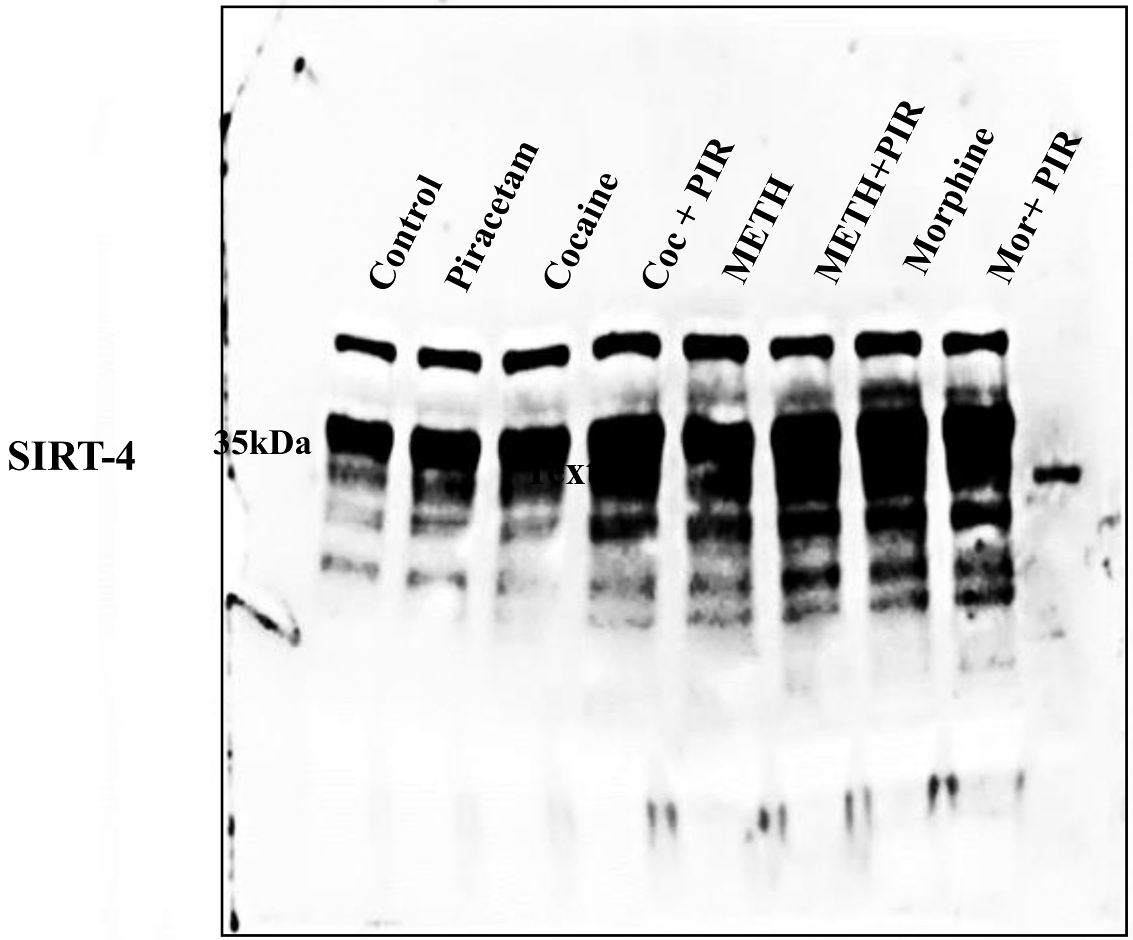

Figure 6 A: Protective Effect of piracetam against psychostimulants and opioids on SIRT-4 in human primary astrocytes.
The representative blot shows SIRT-4 protein level in control, cocaine (1 µM), METH (10 µM) and morphine (5 µM) alone or in combination with piracetam (10 µM)

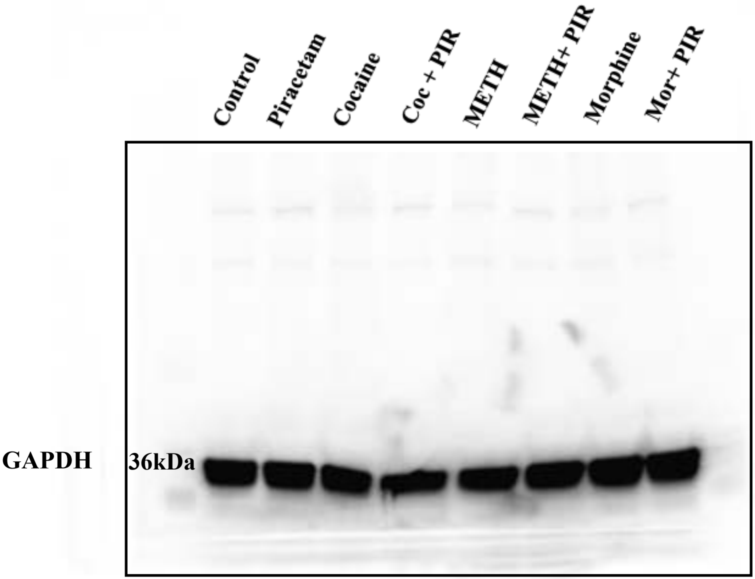

Figure 6 A: GAPDH for SIRT-4 in human primary astrocytes.
The representative blot shows GAPDH for SIRT-4 in control, cocaine (1 µM), METH (10 µM) and morphine (5 µM) alone or in combination with piracetam (10 µM)

Figure 6

B

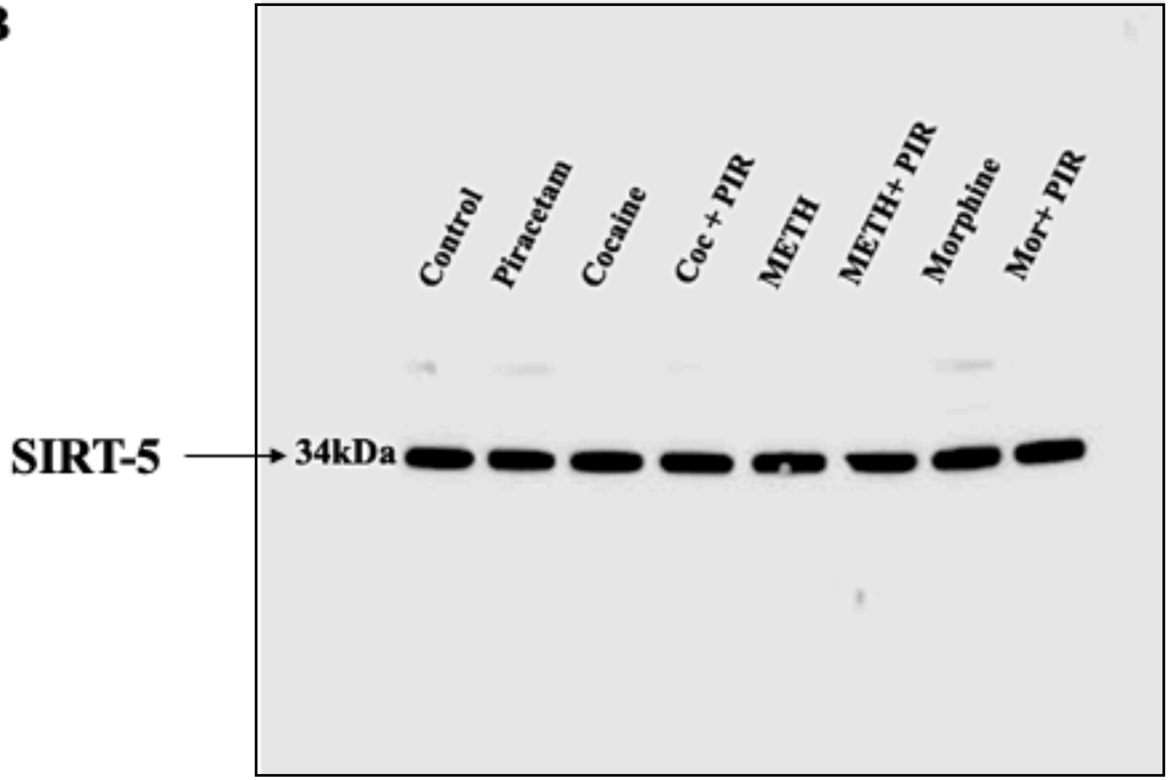

Figure 6 B: Protective Effect of piracetam against psychostimulants and opioids on SIRT-5 in human primary astrocytes.
The representative blot shows SIRT-5 protein level in control, cocaine (1 µM), METH (10 µM) and morphine (5 µM) alone or in combination with piracetam (10 µM)

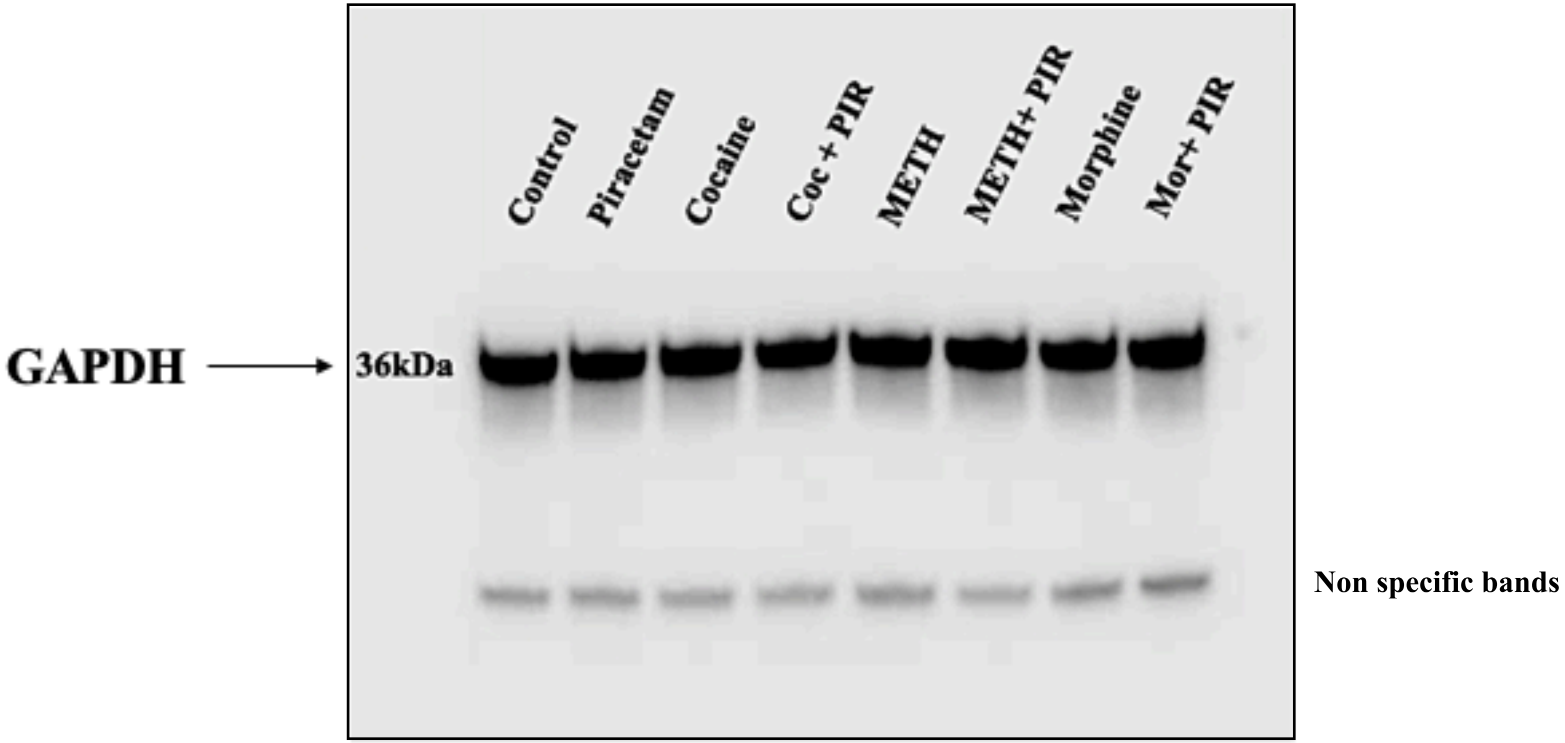

Figure 6 B: GAPDH for SIRT-5 in human primary astrocytes.
The representative blot shows GAPDH for SIRT-5 in control, cocaine (1 µM), METH (10 µM) and morphine (5 µM) alone or in combination with piracetam (10 µM)

Figure 6

E

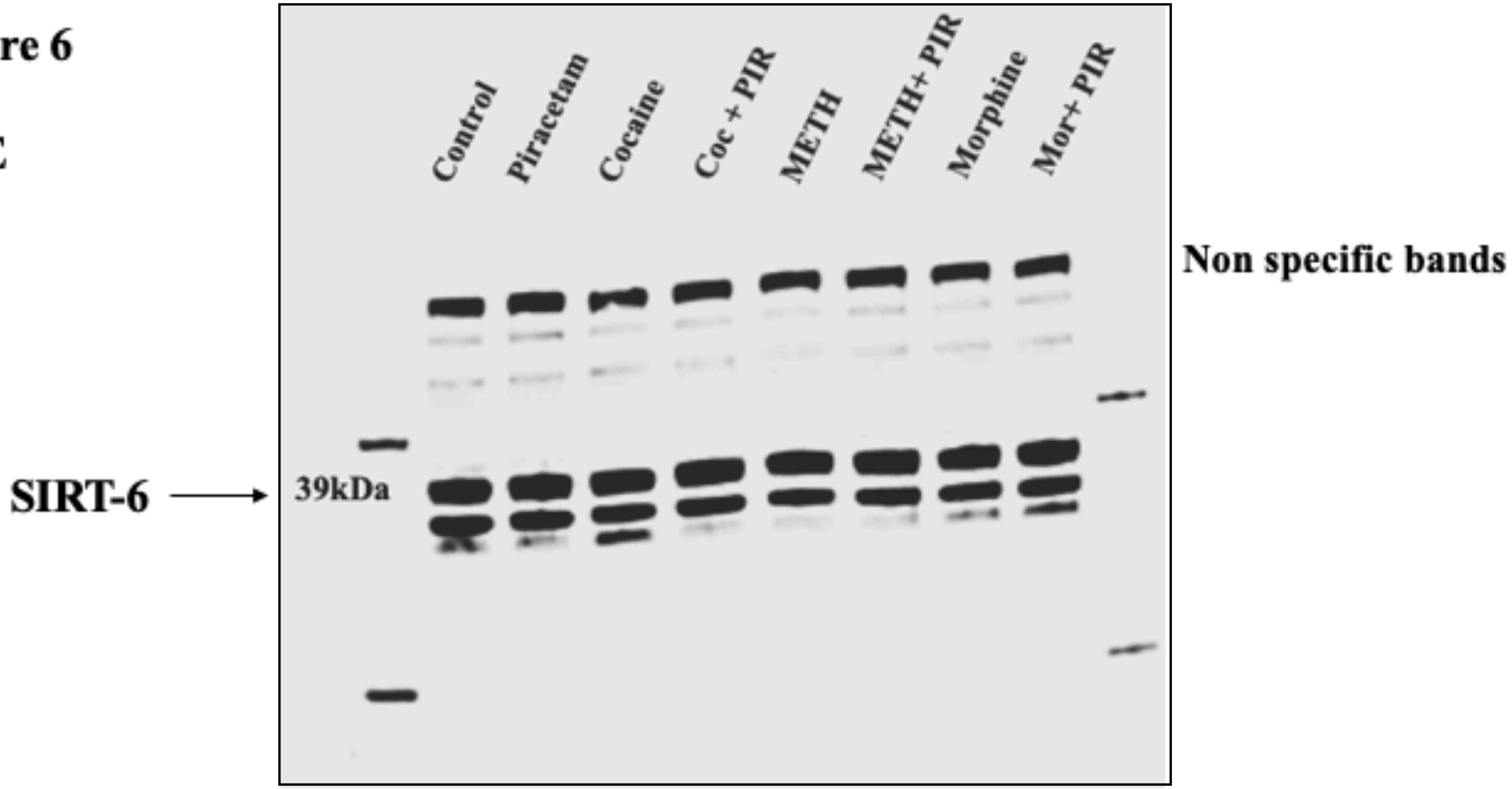

Figure 6 E: Protective Effect of piracetam against psychostimulants and opioids on SIRT-6 in human primary astrocytes.

The representative blot shows SIRT-6 protein level in control, cocaine (1 µM), METH (10 µM) and morphine (5 µM) alone or in combination with piracetam (10 µM)

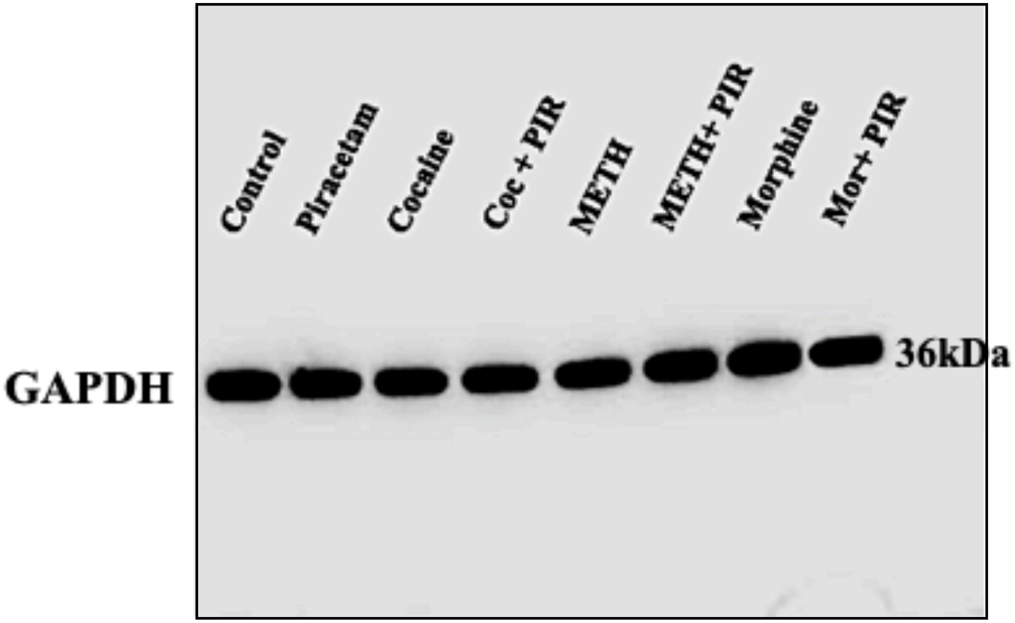

Figure 6 E: GAPDH for SIRT-6 in human primary astrocytes.
The representative blot shows GAPDH for SIRT-6 in control, cocaine (1 µM), METH (10 µM) and morphine (5 µM) alone or in combination with piracetam (10 µM)

Figure 6

F

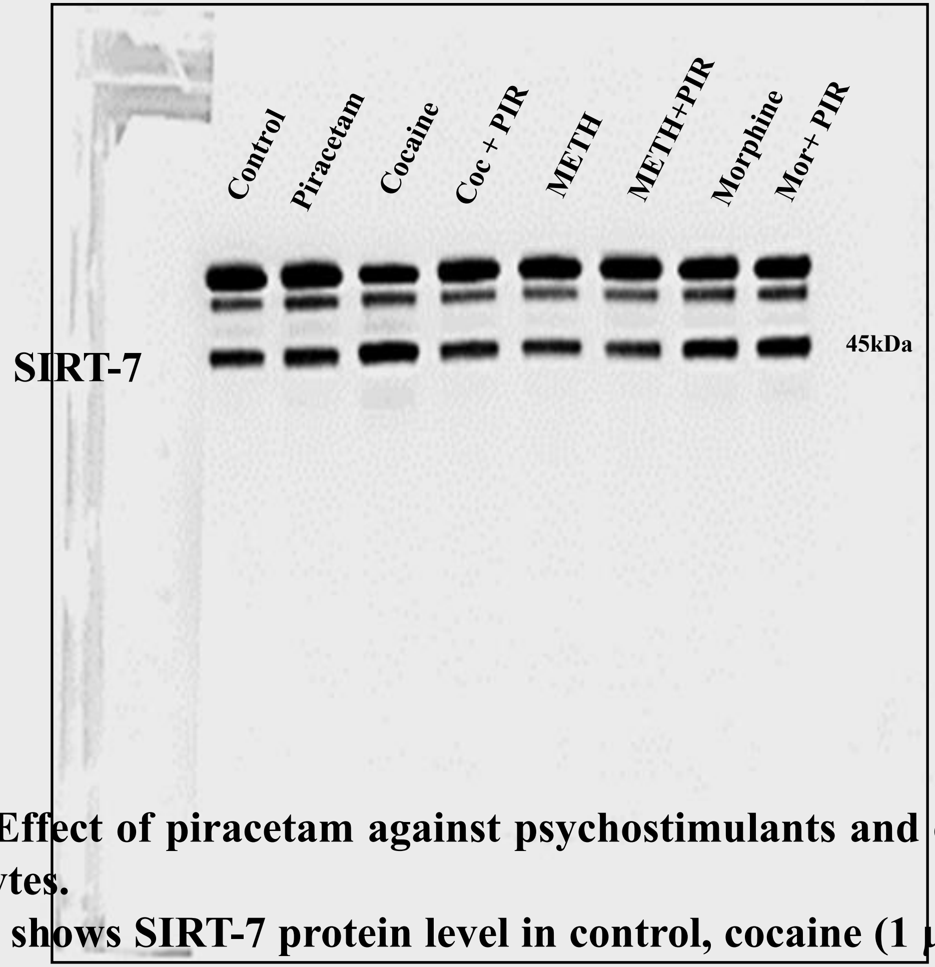

Figure 6 F: Protective Effect of piracetam against psychostimulants and opioids on SIRT-7 in human primary astrocytes.
The representative blot shows SIRT-7 protein level in control, cocaine (1 µM), METH (10 µM) and morphine (5 µM) alone or in combination with piracetam (10 µM)

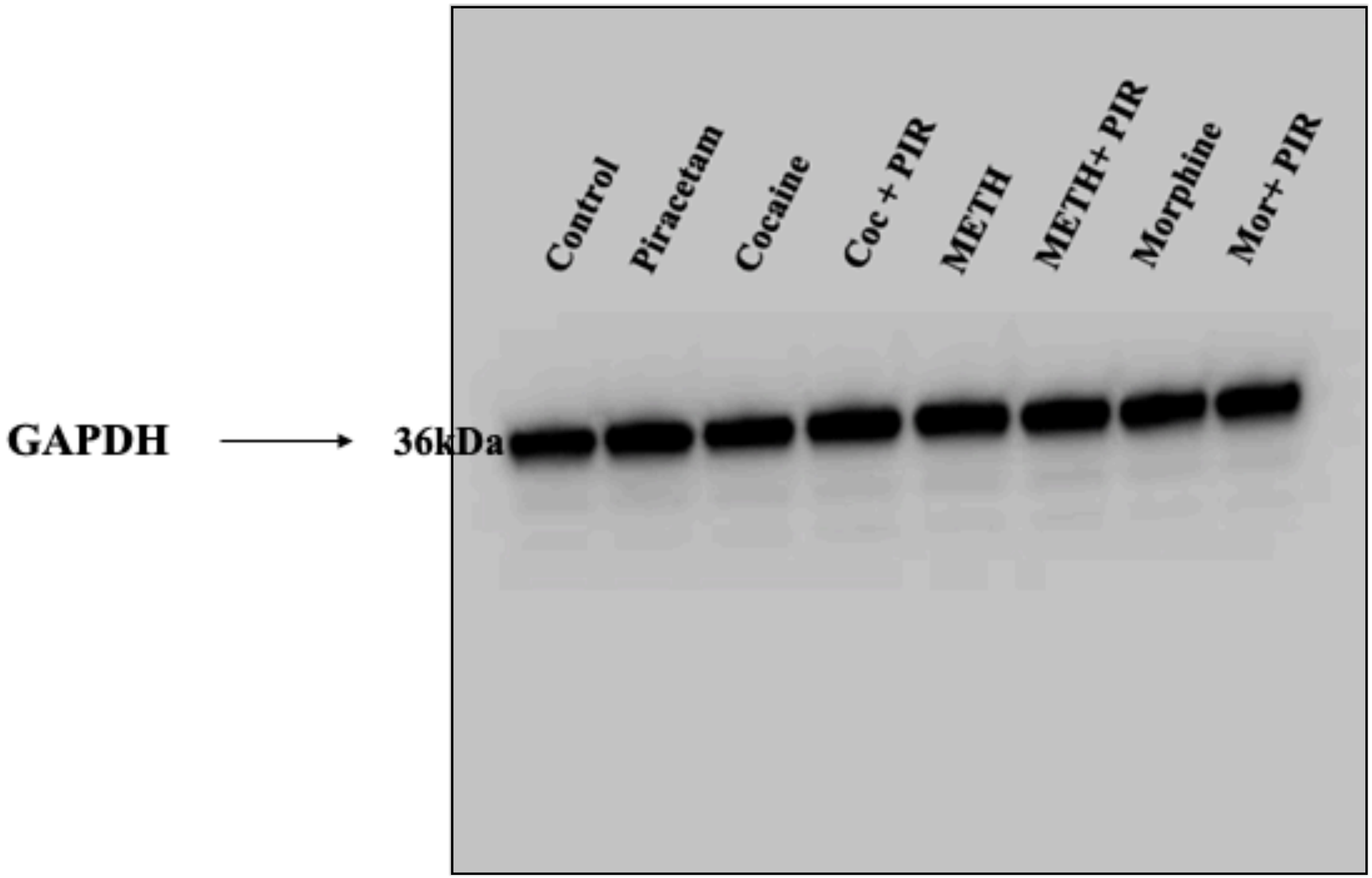

Figure 6 F: GAPDH for SIRT-7 in human primary astrocytes.
The representative blot shows GAPDH for SIRT-7 in control, cocaine (1 µM), METH (10 µM) and morphine (5 µM) alone or in combination with piracetam (10 µM)
