# Supplementary material for: Influence of psychostimulants and opioids on epigenetic modification of class III histone deacetylase (HDAC)-sirtuins in glial cells
Source: Sci Rep. 2021 Oct 29;11:21335. doi: 10.1038/s41598-021-00836-z (PMC8556237; doi:10.1038/s41598-021-00836-z)
Supplement: Supplementary file 6 — Supplementary Information 6. [file 41598_2021_836_MOESM6_ESM.pdf]

Figure 7

A

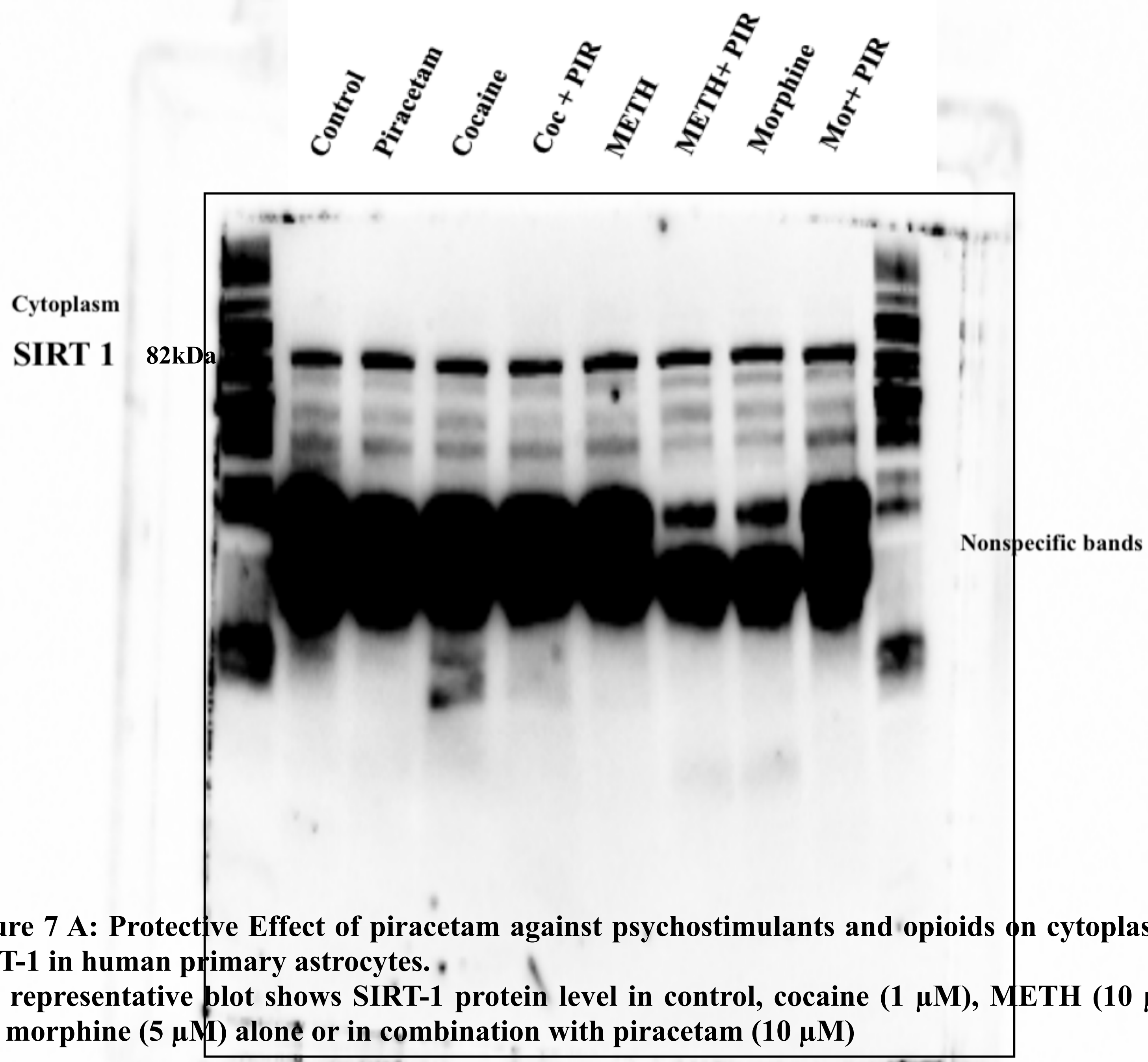

Figure 7 A: Protective Effect of piracetam against psychostimulants and opioids on cytoplasmic SIRT-1 in human primary astrocytes.

The representative blot shows SIRT-1 protein level in control, cocaine (1 µM), METH (10 µM) and morphine (5 µM) alone or in combination with piracetam (10 µM)

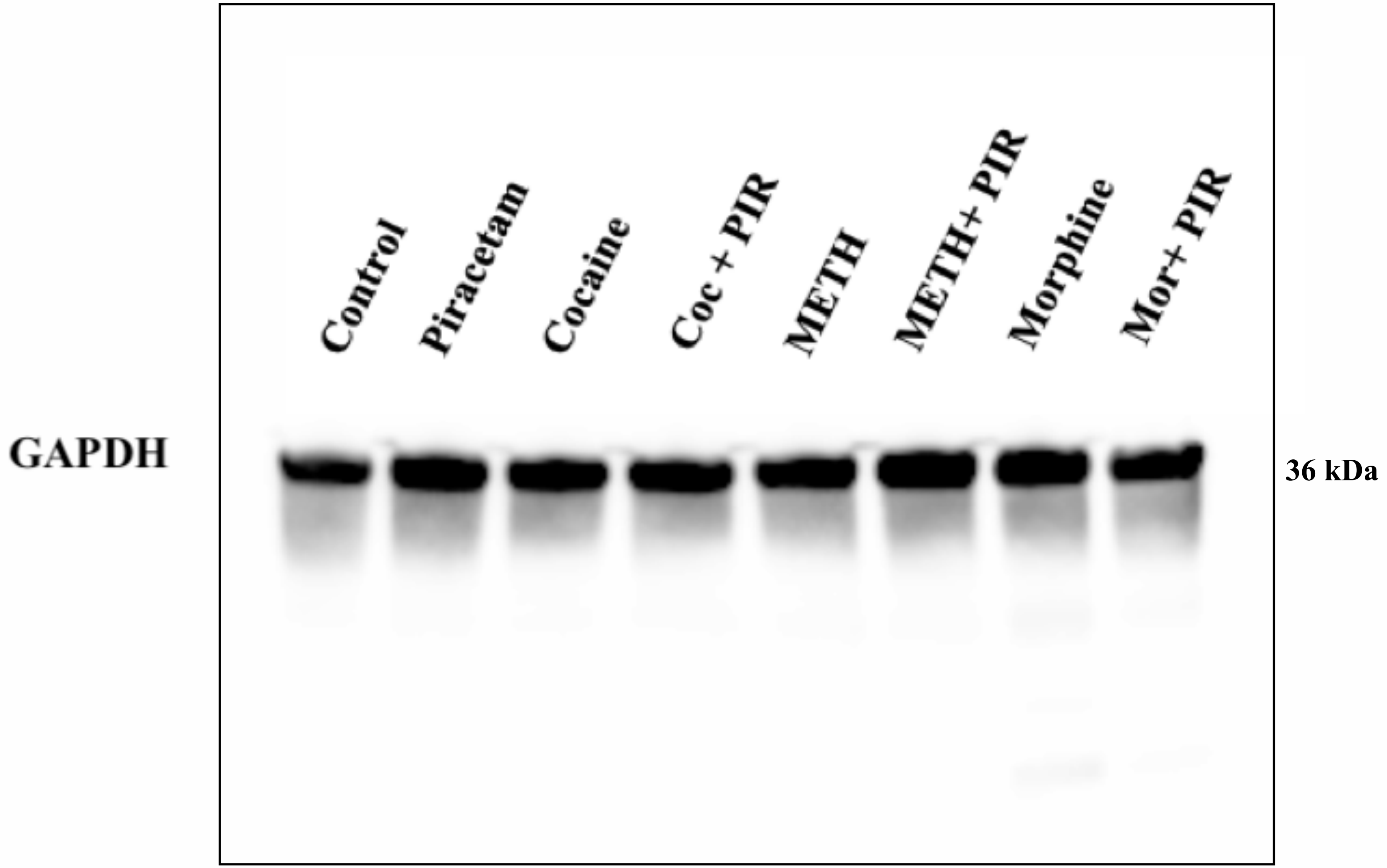

Figure 7 A: GAPDH for cytoplasmic SIRT-1 in human primary astrocytes.
The representative blot shows GAPDH for SIRT-4 in control, cocaine (1 µM), METH (10 µM) and morphine (5 µM) alone or in combination with piracetam (10 µM)

Figure 7

C

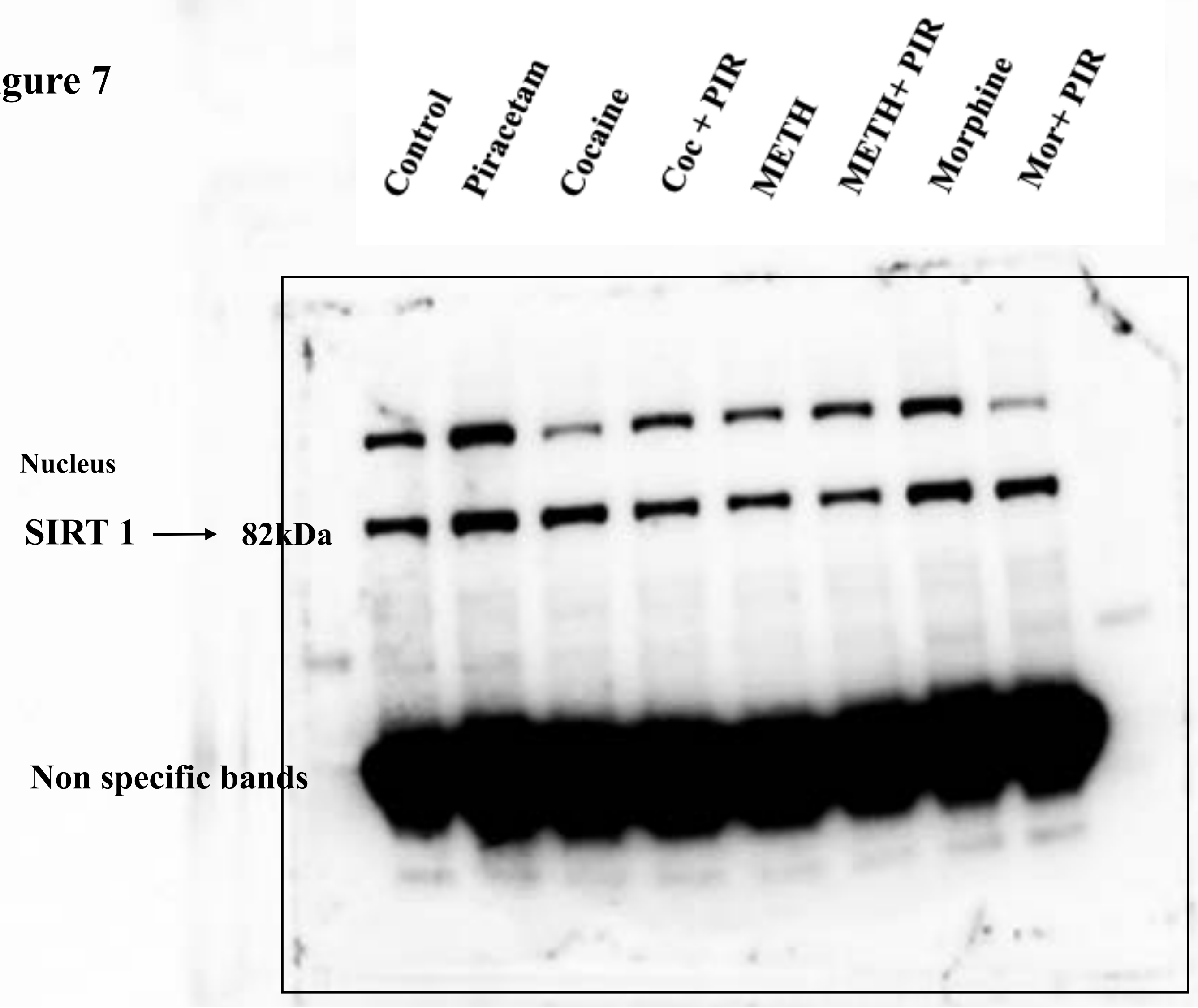

Figure 7 C: Protective Effect of piracetam against psychostimulants and opioids on nuclear SIRT-1 in human primary astrocytes.
The representative blot shows SIRT-1 protein level in control, cocaine (1 µM), METH (10 µM) and morphine (5 µM) alone or in combination with piracetam (10 µM)

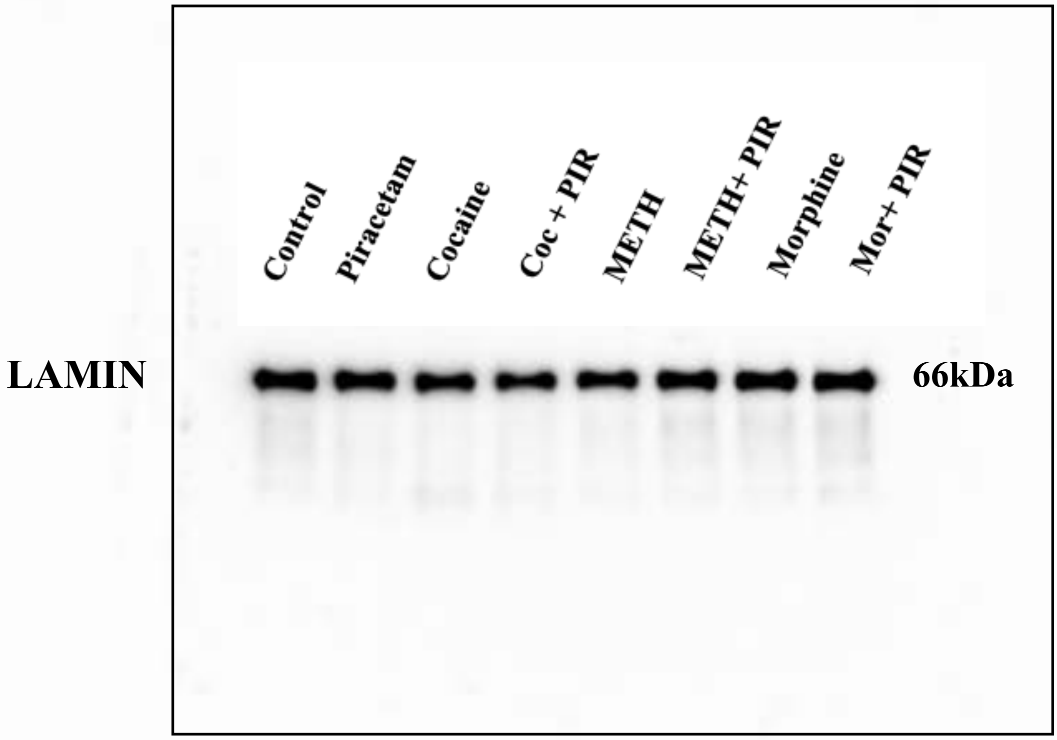

Figure 7 C: LAMIN for nuclear SIRT-1 in human primary astrocytes. The representative blot shows LAMIN for SIRT-1 in control, cocaine (1 μM), METH (10 μM) and morphine (5 μM) alone or in combination with piracetam (10 μM)

Figure 7

E

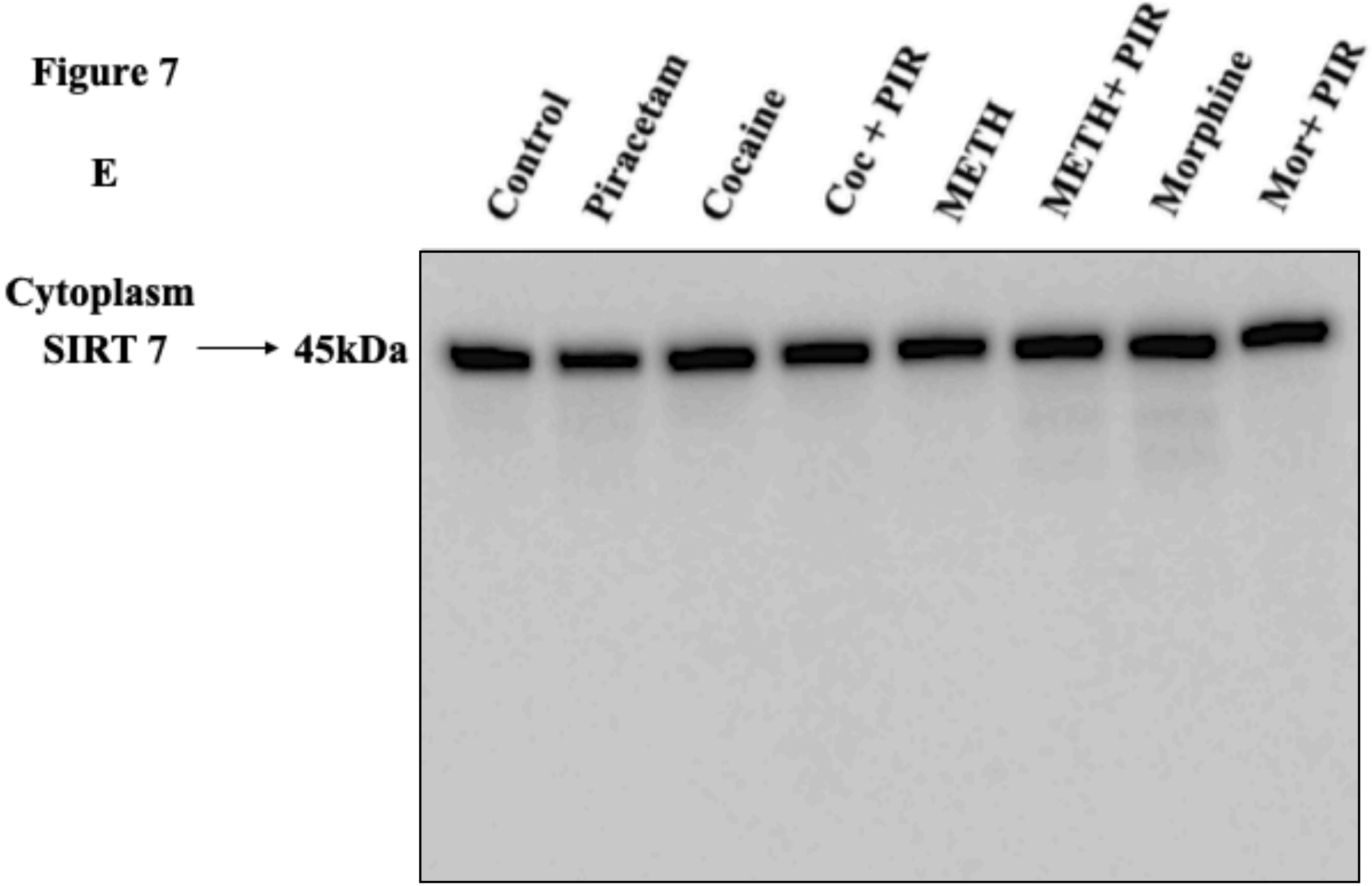

Figure 7 E: Protective Effect of piracetam against psychostimulants and opioids on cytoplasmic SIRT-7 in human primary astrocytes.
The representative blot shows SIRT-7 protein level in control, cocaine (1 µM), METH (10 µM) and morphine (5 µM) alone or in combination with piracetam (10 µM)

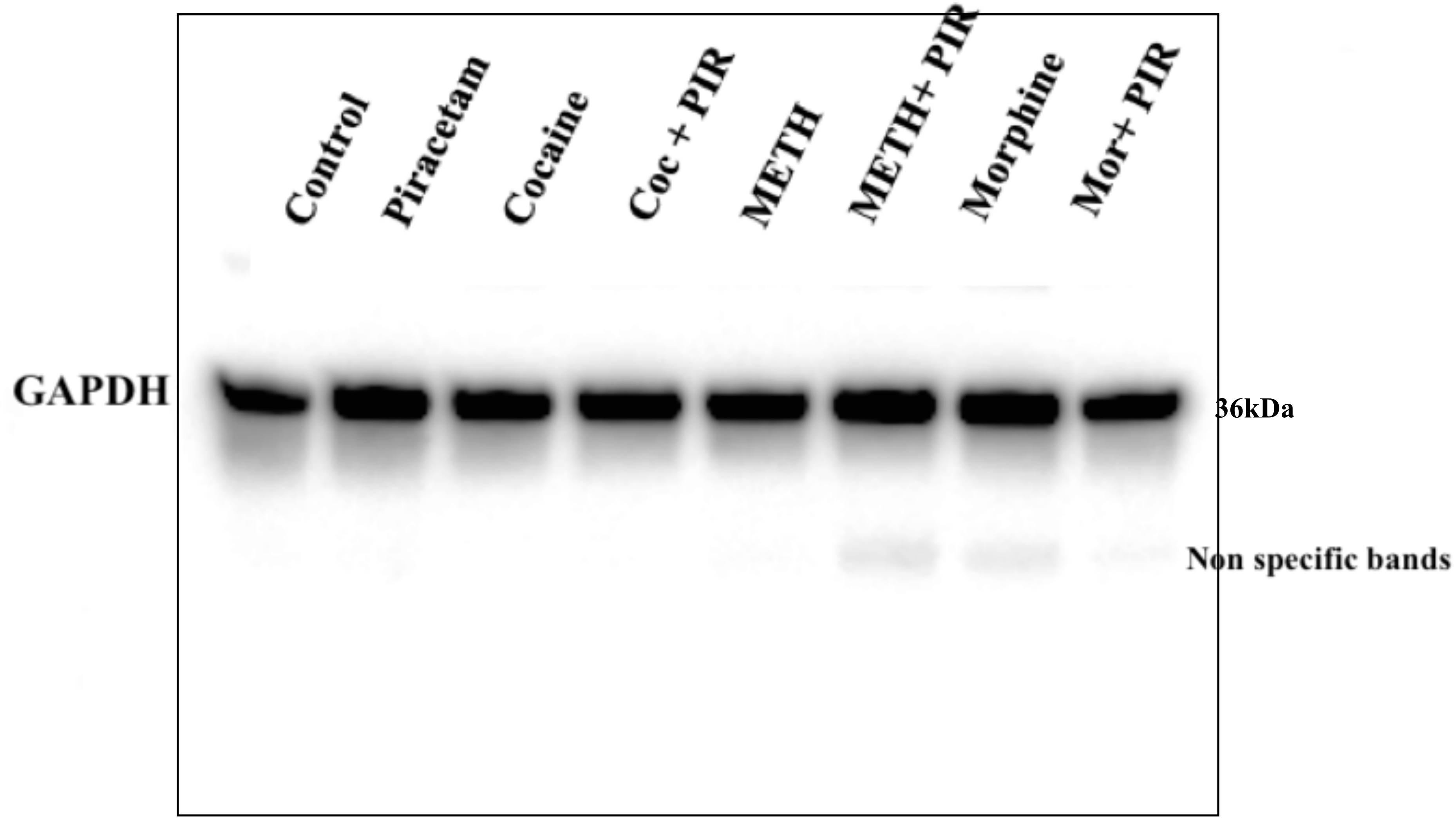

Figure 7 E: GAPDH for cytoplasmic SIRT-7 in human primary astrocytes.
The representative blot shows GAPDH for SIRT-7 in control, cocaine (1 µM), METH (10 µM) and morphine (5 µM) alone or in combination with piracetam (10 µM)

Figure 7

G

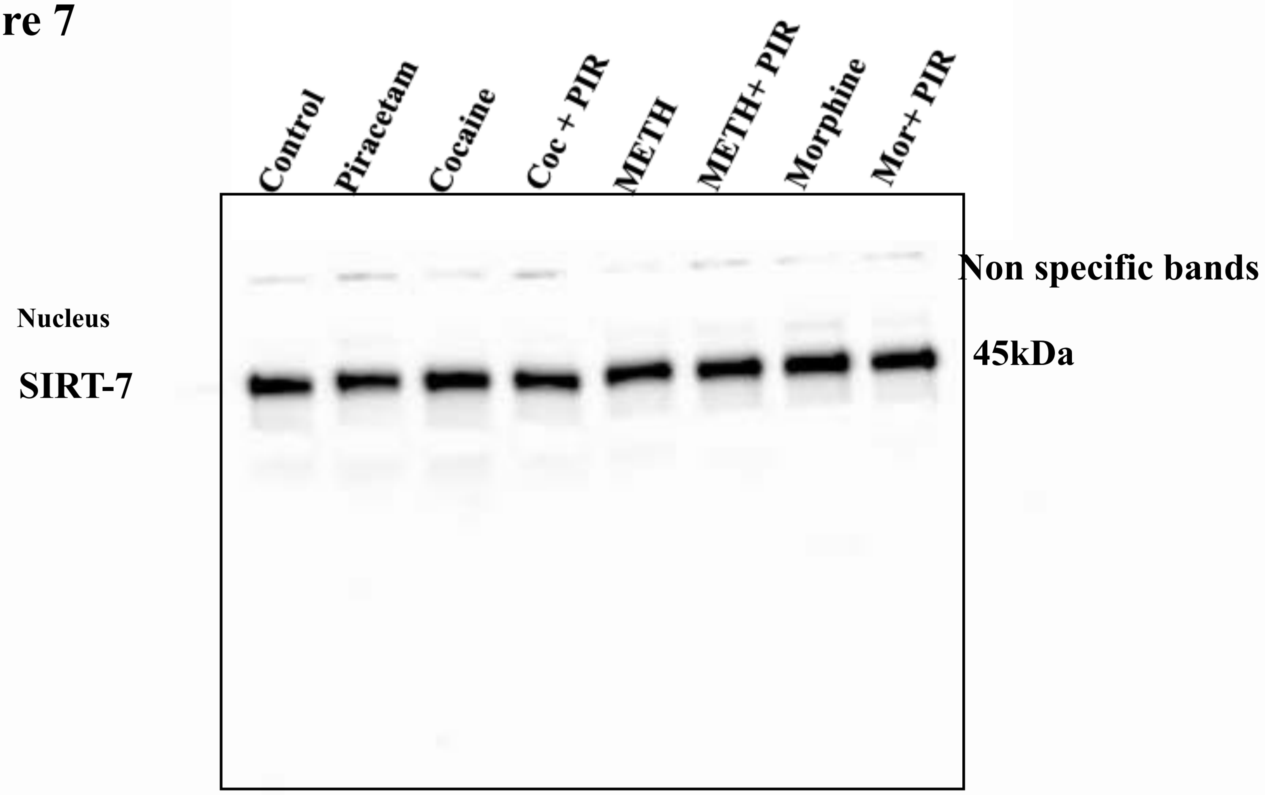

Figure 7 G: Protective Effect of piracetam against psychostimulants and opioids on nuclear SIRT-7 in human primary astrocytes.

The representative blot shows SIRT-7 protein level in control, cocaine (1 µM), METH (10 µM) and morphine (5 µM) alone or in combination with piracetam (10 µM)

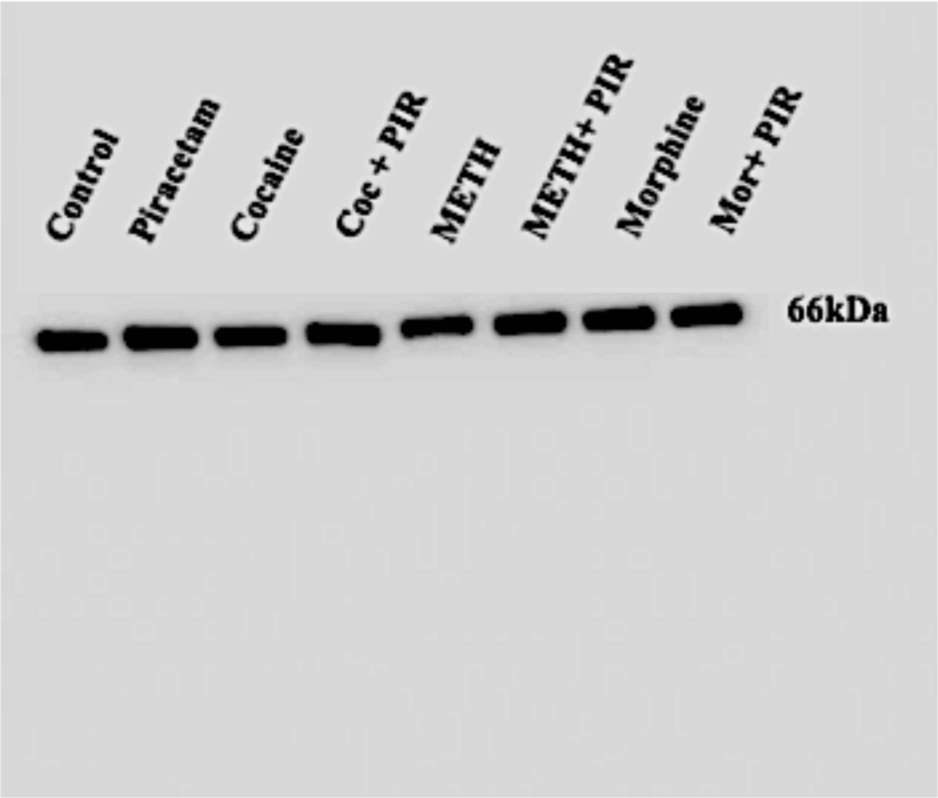

Figure 7 G: LAMIN for nuclear SIRT-7 in human primary astrocytes. The representative blot shows LAMIN for SIRT-7 in control, cocaine (1 µM), METH (10 µM) and morphine (5 µM) alone or in combination with piracetam (10 µM)
